# Stability analysis for a class of implicit fractional differential equations involving Atangana–Baleanu fractional derivative

**DOI:** 10.1186/s13662-021-03551-1

**Published:** 2021-08-24

**Authors:** Asma, Sana Shabbir, Kamal Shah, Thabet Abdeljawad

**Affiliations:** 1 Department of Mathematics, COMSATS University of Islamabad, Sahiwal Campus, Punjab, Pakistan; 2 Department of Mathematics, University of Malakand, Dir(L), Khyber Pakhtunkhwa, Pakistan; 3 Department of Mathematics and General Sciences, Prince Sultan University, Riyadh, Saudi Arabia; 4 Department of Medical Research, China Medical University, Taichung, 40402 Taiwan; 5 Department of Computer Science and Information Engineering, Asia University, Taichung, Taiwan

**Keywords:** 26A33, 34A08, 93A30, Atangana–Baleanu–Caputo derivative, Stability results, Fixed point theorems

## Abstract

Some fundamental conditions and hypotheses are established to ensure the existence, uniqueness, and stability to a class of implicit boundary value problems (BVPs) with Atangana–Baleanu–Caputo type derivative and integral. The required results are established by utilizing the Banach contraction mapping principle and fixed point theorem of Krasnoselskii. In addition, various types of stability results including Hyers–Ulam, generalized Hyers–Ulam, Hyers–Ulam–Rassias, and generalized Hyers–Ulam–Rassias stability are formulated for the problem under consideration. Pertinent examples are given to justify the results we obtain.

## Introduction

Our world is a combination of scientific phenomena. Mathematics is the mother of support to all sciences. Our many real life problems and many natural phenomena depend on or are governed by mathematical laws. Certain real life problems can be modeled and deeply studied by using calculus methods of integration and derivative. However, many problems can be studied more accurately by using fractional calculus methods. That is why fractional differential equations (FDEs) have become a major concern for many well-renowned researchers and scientists. A fractional derivative of arbitrary order can be real or even a complex number as well. The credit for the first thought about fractional derivative goes to L. Hopital and Leibnitz in 1695 [1]. After this initial step, the researchers in this area kept on flourishing because of its numerous applications in physics, economics, engineering, and biological sciences.

In the last few years, FDEs got more attention. Many researchers also examined the solutions of fractional models for stability analysis [2]. Some researchers also worked on COVID-19 model with the help of fractional calculus [3, 4]. On the other hand, fixed point theory helps much in the development of the qualitative and computational concepts of FDEs and their related dynamic equations [5–11]. But still no fixed definition of fractional operators has been defined. Researchers have worked on it rigorously and given many definitions with singular or nonsingular kernels. A lot of work has been done using Riemann–Liouville and Caputo fractional derivative and integration. After this Caputo and Fabrizio worked together and gave a new definition named Caputo–Fabrizio derivative. This derivative has a nonsingular kernel. After Caputo, Fabrizio, Atangana, and Baleanu gave another definition known as $\mathcal{AB}$ derivative. The $\mathcal{AB}$ derivative involves a nonlocal and nonsingular kernel [12]. The $\mathcal{AB}$ derivative, due to its Mittag-Leffler type kernel, has produced more interesting results as compared to Caputo–Fabrizio derivative whose exponential kernel can be splitter and hence affects the delay action. The new formulation of Caputo derivative by Atangana and Baleanu is known as Atangana–Baleanu–Caputo ($\mathcal{ABC}$) and Riemann–Liouville ($\mathcal{ABR}$) derivatives [13]. Recently a significant contribution has been made by various authors and the field of FDEs (for details, see [11, 14–17]).

Pantograph type FDEs play an important role in physics and applied mathematics. That’s why these pantograph type delay FDEs have been deeply studied by many researchers. Jarad and his coauthors studied FDEs with Atangana–Baleanu–Caputo $(\mathcal{ABC})$ derivative [18] as follows:

1 $$\begin{aligned} \textstyle\begin{cases} {}_{\mathrm{a}}^{\mathcal{ABC}}\mathcal{D}^{\mathrm{\alpha }}_{ \mathrm{t}} \mathrm{u}(\mathrm{t}) = \mathrm{g}(\mathrm{t}, \mathrm{u}(\mathrm{t})),\quad 0< \mathrm{\alpha }\leq 1, \mathrm{t}\in [\mathrm{a}, \mathrm{b}], \\ \mathrm{u}(\mathrm{a})= \mathrm{u}_{\mathrm{a}}. \end{cases}\displaystyle \end{aligned}$$

Abdo and his coauthors worked on the following nonlinear pantograph FDEs [13, 19]:

2 $$\begin{aligned} \textstyle\begin{cases} {}_{\mathrm{a}}^{\mathcal{ABC}}\mathcal{D}^{\mathrm{\alpha }}_{ \mathrm{t}}\mathrm{u}(\mathrm{t}) = \mathrm{g}(\mathrm{t}, \mathrm{u}(\mathrm{t}), \mathrm{u}(\gamma \mathrm{t})), \quad 0< \mathrm{\alpha }\leq 1, \mathrm{t}\in [\mathrm{a}, \mathrm{b}], \\ \mathrm{u}(\mathrm{a})=\Sigma _{k=1}^{m}c_{k}\mathrm{u}(\mathrm{t}_{k}), \quad \mathrm{t}_{k}\in (\mathrm{a}, \mathrm{b}), \end{cases}\displaystyle \end{aligned}$$

where $0<\mathrm{\alpha }\leq 1 $ is the order of derivative ${}_{\mathrm{a}}^{\mathcal{ABC}}\mathcal{D}^{\mathfrak{\mathrm{\alpha }}}_{ \mathrm{t}}$, $\mathrm{g}:[\mathrm{a}, \mathrm{b}]\times \mathcal{R} \times \mathcal{R}\longrightarrow \mathcal{R}$ is a continuous function, and $\gamma \in (0,1]$.

In the last few years the area devoted to investigating initial and BVPs under $\mathcal{ABC}$-fractional order derivative has been developed very well. Here, we remark that the area devoted to studying biological models under the said derivative has been investigated very well. Numerical interpretations for various kinds of FDEs under the aforesaid derivative have been studied very well in the last few years [20]. Inspired by the research work as mentioned above, we intend to work on a implicit BVP involving $(\mathcal{ABC})$ derivative of the form:

3 $$\begin{aligned} \textstyle\begin{cases} {} _{0}^{\mathcal{ABC}}\mathcal{\mathcal{D}}^{\mathrm{\alpha }}_{ \mathrm{t}}\mathrm{u}(\mathrm{t}) = {\mathrm{g}}(\mathrm{t}, \mathrm{u}(\mathrm{t}),{}_{0}^{\mathcal{ABC}}\mathcal{D}^{ \mathrm{\alpha }}_{\mathrm{t}}\mathrm{u} (\mathrm{t})), \quad 1< \mathrm{\alpha }\leq 2, \mathrm{t}\in \mathcal{J}=[0, \mathrm{b}], \\ \mathrm{u}(0) = \mathrm{u}_{0},\qquad \mathrm{u}( \mathfrak{{\mathrm{b}}})=\mathrm{u}_{1}, \end{cases}\displaystyle \end{aligned}$$

where ${}_{0}^{\mathcal{ABC}}\mathcal{\mathcal{D}}^{\mathrm{\alpha }}_{ \mathrm{t}} $ represents the $\mathcal{ABC}$ derivative of order $1 <\mathrm{\alpha } \leq 2 $ and $\mathrm{g}: \mathcal{J} \times \mathcal{R} \times \mathcal{R} \longrightarrow \mathcal{R} $ is a continuous function.

We compute results for implicit FDEs with boundary conditions. Most of our derivations are made using theorems of significant importance such as Krasnoselskii, Arzelá–Ascoli and Banach fixed point theorems. Also we derive various results for Ulam–Hyers type stability analysis of our problem as the said stability has been investigated in the last few years very well in respect of FDEs. Also some researchers have investigated various results of the concerned stability in nonlinear analysis (see [21–25]).

## Fundamental results

Here, we recollect some basic definitions of fractional calculus which are needed throughout this work. We derive our main results by using these fundamental results. Suppose that $\mathbb{X}=\mathcal{C}[\mathcal{J}, \mathcal{R}]$ is a Banach space and the norm under consideration is $\Vert \mathrm{u}\Vert = \max_{\mathrm{t} \in \mathcal{J}} | \mathrm{u}(\mathrm{t})|$.

### Definition 1

([26, 27] $\mathcal{ABC}\text{ fractional derivative}$)

Let $\mathrm{u}\in \mathrm{H}^{1} (c, d)$, $c < d $, and $\mathfrak{\mathrm{\alpha }} \in (0, 1)$. The ${\mathcal{ABC}}$ fractional derivative for function u of order *α* is defined as

4 $$\begin{aligned} {}_{0}^{{\mathcal{ABC}}}\mathcal{D}^{\mathfrak{\mathrm{\alpha }}}_{ \mathrm{t}} \mathrm{u}(\mathrm{t}) = \frac{\mathbb{M} ({\mathfrak{\mathrm{\alpha }}})}{1- {\mathfrak{\mathrm{\alpha }}}} \int ^{\mathrm{t}}_{0} \mathrm{u}^{\prime }(\xi ) \mathrm{E}_{ \mathfrak{\mathrm{\alpha }}} \biggl( \frac{-{\mathfrak{\mathrm{\alpha }}}(\mathrm{t}-\xi )^{\mathfrak{\mathrm{\alpha }}}}{1 - {\mathfrak{\mathrm{\alpha }}}} \biggr)\,d\xi. \end{aligned}$$

We use this definition throughout the paper. Further, the mentioned operator in the Riemann–Liouville sense is defined as

5 $$\begin{aligned} {}_{0}^{{\mathcal{ABR}}}\mathcal{D}^{\mathfrak{\mathrm{\alpha }}}_{ \mathrm{t}} \mathrm{u}(\mathrm{t})= \frac{\mathbb{M}({\mathfrak{\mathrm{\alpha }}})}{1-{\mathfrak{\mathrm{\alpha }}}} \frac{d}{d\mathrm{t}} \int ^{\mathrm{t}}_{0}\mathrm{u}(\xi ) \mathrm{E}_{\mathfrak{\mathrm{\alpha }}} \biggl( \frac{-{\mathfrak{\mathrm{\alpha }}}(\mathrm{t}-\xi )^{\mathfrak{\mathrm{\alpha }}}}{1-{\mathfrak{\mathrm{\alpha }}}} \biggr)\,d\xi. \end{aligned}$$

In the above equations $\mathbb{M}(\mathfrak{\mathrm{\alpha }})>0$ is a normalization function. Here, $\mathbb{M}(0) = \mathbb{M}(1) = 1 $ and $\mathrm{E}_{\mathfrak{\mathrm{\alpha }}} $ represents the well-known Mittag-Leffler function.

### Definition 2

([26, 27] $\mathcal{AB}\text{ fractional integral}$)

Let u be a function, then the ${\mathcal{AB}}$ fractional integral of order ${\mathfrak{\mathrm{\alpha }}} \in (0, 1) $ is defined by

6 $$\begin{aligned} {}_{0}^{\mathcal{AB}}\mathcal{I}^{\mathfrak{\mathrm{\alpha }}}_{ \mathrm{t}} \mathrm{u}(\mathrm{t}) = \frac{1-{\mathfrak{\mathrm{\alpha }}}}{\mathbb{M}({\mathfrak{\mathrm{\alpha }}})} \mathrm{u}(\mathrm{t}) + \frac{\mathfrak{\mathrm{\alpha }}}{\mathbb{M}({\mathfrak{\mathrm{\alpha }}})\Gamma ({\mathfrak{\mathrm{\alpha }}})} \int ^{\mathrm{t}}_{0}\mathrm{u}(\xi ) (\mathrm{t}-\xi )^{{ \mathfrak{\mathrm{\alpha }}}-1}\,d\xi. \end{aligned}$$

### Definition 3

([28])

Let u be a function such that $\mathrm{u}^{(n)}\in \mathrm{H}^{1}(c, d)$ and $n< {\mathfrak{\mathrm{\alpha }}} \leq n+1, n=0,1,\ldots $ . Then the ${\mathcal{ABC}}$ derivative satisfies the following formula:

$$\begin{aligned} _{0}^{{\mathcal{ABC}}}\mathcal{D}^{\mathfrak{\mathrm{\alpha }}}_{ \mathrm{t}}\mathrm{u}( \mathrm{t}) = {}_{0}^{{\mathcal{ABC}}}\mathcal{D}^{ \mathfrak{\beta }}_{\mathrm{t}} \mathrm{u}^{(n)}(\mathrm{t}), \end{aligned}$$

where $\mathfrak{\beta }={\mathfrak{\mathrm{\alpha }}}-n$.

### Lemma 1

([28])

*For* $\mathfrak{\mathrm{\alpha }} \in (n, n+1], n=0,1,2,\ldots$ , *the following outcome holds for the FDEs*:

$$\begin{aligned} _{0}^{\mathcal{AB}}\mathcal{I}^{\mathfrak{\mathrm{\alpha }}}_{ \mathrm{t}}{_{0}^{{\mathcal{ABC}}} \mathcal{D}^{ \mathfrak{\mathrm{\alpha }}} {}_{\mathrm{t}}}\mathrm{u} (\mathrm{t})= \mathrm{u}( \mathrm{t}) + \mathfrak{c}_{0} + \mathfrak{c}_{1} \mathrm{t} + \mathfrak{c}_{2}\mathrm{t}^{2}+\cdots + \mathfrak{c}_{n} \mathrm{t}^{n} \end{aligned}$$

*for an arbitrary constant* $\mathfrak{c}_{i}$ *with* $i = 0, 1, 2,\ldots,n $.

### Theorem 1

([29, 30])

*Let* $\mathcal{W}$ *be a nonempty*, *convex*, *and closed subset of* $\mathbb{X}$. *Consider two operators* $\mathcal{H} $, $\mathcal{B}$ *such that*

1. $\mathcal{H}({w}_{1})+ \mathcal{B}( {w}_{2}) \in \mathcal{W} $ *for all* ${w}_{1}, {w}_{2}\in \mathcal{W} $,
2. $\mathcal{H}$ *is a contraction operator*,
3. $\mathcal{B}$ *is continuous and compact*,

*then there exists at least one solution* ${w}\in \mathbb{X} $, *such that* $\mathcal{H}({w})+\mathcal{B}({w})= {w} $.

## Mathematical analysis of model problem

The present section of our paper is reserved to examining the existence and uniqueness for the solution of our implicit FDEs by Krasnoselskii’s fixed point theorem.

### Lemma 2

*Let* $\varpi \in \mathbb{X} $, *then our problem can be formulated as*

7 $$\begin{aligned} \textstyle\begin{cases} {}{_{0}^{{\mathcal{ABC}}}\mathcal{D}^{\mathfrak{\mathrm{\alpha }}}_{ \mathrm{t}}\mathrm{u}(\mathrm{t})} = \varpi (\mathrm{t}), \quad 1< { \mathfrak{\mathrm{\alpha }}}\leq 2, \mathrm{t}\in \mathcal{J}, \\ \mathrm{u}(0) = \mathrm{u}_{0}, \qquad \mathrm{u}( \mathfrak{\mathrm{b}}) = \mathrm{u}_{1}. \end{cases}\displaystyle \end{aligned}$$

*The solution of the above problem is given by*

$$\begin{aligned} \mathrm{u}(\mathrm{t}) ={}& \frac{\mathrm{t}\mathrm{u}_{1} + \mathrm{u}_{0}(\mathfrak{\mathrm{b}}-\mathrm{t})}{\mathfrak{\mathrm{b}}}- \frac{\mathrm{t}(2-{\mathfrak{\mathrm{\alpha }}})}{\mathfrak{\mathrm{b}} \mathbb{M}({\mathfrak{\mathrm{\alpha }}}-1)} \int _{0}^{\mathfrak{\mathrm{b}}}\varpi (\xi )\,d\xi \\ &{}- \frac{\mathrm{t}({\mathfrak{\mathrm{\alpha }}}-1)}{\mathfrak{\mathrm{b}}\mathbb{M}({\mathfrak{\mathrm{\alpha }}}-1)\Gamma ({\mathfrak{\mathrm{\alpha }}})} \int _{0}^{\mathfrak{\mathrm{b}}}\varpi (\xi ) (\mathfrak{\mathrm{b}}- \xi )^{{\mathfrak{\mathrm{\alpha }}}-1}\,d\xi \\ &{}+ \frac{2-{\mathfrak{\mathrm{\alpha }}}}{\mathbb{M}({\mathfrak{\mathrm{\alpha }}}-1)} \int _{0}^{\mathrm{t}}\varpi (\xi )\,d\xi + \frac{{\mathfrak{\mathrm{\alpha }}}-1}{\mathbb{M}({\mathfrak{\mathrm{\alpha }}}-1)\Gamma ({\mathfrak{\mathrm{\alpha }}})} \int _{0}^{\mathrm{t}}\varpi (\xi ) (\mathrm{t}-\xi )^{{ \mathfrak{\mathrm{\alpha }}}-1}\,d\xi. \end{aligned}$$

### Proof

Consider ${_{0}^{{\mathcal{ABC}}}\mathcal{D}^{\mathfrak{\mathrm{\alpha }}}_{ \mathrm{t}}\mathrm{u}(\mathrm{t})} = \varpi (\mathrm{t})$. Then, by applying the integral ${}_{0}^{\mathcal{AB}}\mathcal{I}_{\mathrm{t}}^{ \mathfrak{\mathrm{\alpha }}}$ on both sides, we get

8 $$\begin{aligned} \mathrm{u}(\mathrm{t})= \mathfrak{ c}_{0}+ \mathfrak{c}_{1}\mathrm{t}+ \frac{2-{\mathfrak{\mathrm{\alpha }}}}{\mathbb{M}({\mathfrak{\mathrm{\alpha }}}-1)} \int _{0}^{\mathrm{t}}\varpi (\xi )\,d\xi + \frac{{\mathfrak{\mathrm{\alpha }}}-1}{\mathbb{M}({\mathfrak{\mathrm{\alpha }}}-1) \Gamma ({\mathfrak{\mathrm{\alpha }}})} \int _{0}^{\mathrm{t}}\varpi ( \xi ) (\mathrm{t}-\xi )^{{\mathfrak{\mathrm{\alpha }}}-1}\,d\xi. \end{aligned}$$

Now, using the boundary condition $\mathrm{u}(0) = \mathrm{u}_{0} $, we get $\mathfrak{c}_{0} = \mathrm{u}_{0}$.

Now, for $\mathrm{u}(\mathrm{b}) = \mathrm{u}_{1}$, we get

$$\begin{aligned} \mathfrak{c}_{1}= \frac{\mathrm{u}_{1}-\mathrm{u}_{0}}{{\mathrm{b}}} - \frac{2-{\mathfrak{\mathrm{\alpha }}}}{{\mathrm{b}}\mathbb{M}({\mathfrak{\mathrm{\alpha }}}-1)} \int _{0}^{{\mathrm{b}}} \varpi (\xi )\,d\xi - \frac{{\mathfrak{\mathrm{\alpha }}}-1}{\mathrm{b}\mathbb{M}({\mathfrak{\mathrm{\alpha }}}-1)\Gamma ({\mathfrak{\mathrm{\alpha }}})} \int _{0}^{{\mathrm{b}}} \varpi (\xi ) ({\mathrm{b}}-\xi )^{{ \mathfrak{\mathrm{\alpha }}}-1}\,d\xi. \end{aligned}$$

Substituting the values of $\mathfrak{c}_{0}$ and $\mathfrak{c}_{1}$ in equation (8), we get

$$\begin{aligned} \mathrm{u}(\mathrm{t}) ={}& \frac{\mathrm{t}\mathrm{u}_{1} + \mathrm{u}_{0}({\mathrm{b}}-\mathrm{t})}{{\mathrm{b}}}- \frac{\mathrm{t}(2-{\mathfrak{\mathrm{\alpha }}})}{{\mathrm{b}} \mathbb{M}({\mathfrak{\mathrm{\alpha }}}-1)} \int _{0}^{{\mathrm{b}}}\varpi (\xi )\,d\xi \\ & {}- \frac{\mathrm{t}({\mathfrak{\mathrm{\alpha }}}-1)}{{\mathrm{b}}\mathbb{M}({\mathfrak{\mathrm{\alpha }}}-1) \Gamma ({\mathfrak{\mathrm{\alpha }}})} \int _{0}^{{\mathrm{b}}} \varpi (\xi ) ({\mathrm{b}}-\xi )^{{\mathfrak{\mathrm{\alpha }}}-1}\,d \xi \\ &{}+ \frac{2-{\mathfrak{\mathrm{\alpha }}}}{\mathbb{M}({\mathfrak{\mathrm{\alpha }}}-1)} \int _{0}^{\mathrm{t}}\varpi (\xi )\,d\xi + \frac{{\mathfrak{\mathrm{\alpha }}}-1}{\mathbb{M}({\mathfrak{\mathrm{\alpha }}}-1)\Gamma ({\mathfrak{\mathrm{\alpha }}})} \int _{0}^{\mathrm{t}}\varpi (\xi ) (\mathrm{t}-\xi )^{{ \mathfrak{\mathrm{\alpha }}}-1}\,d\xi. \end{aligned}$$

 □

### Corollary 1

*In view of Lemma *2, *our problem* (3) *is equivalent to the following integral equation*:

$$\begin{aligned} \mathrm{u}(\mathrm{t}) ={}& \frac{\mathrm{t}\mathrm{u}_{1} + \mathrm{u}_{0}({\mathrm{b}}-\mathrm{t})}{{\mathrm{b}}}- \frac{\mathrm{t}(2-{\mathfrak{\mathrm{\alpha }}})}{{\mathrm{b}} \mathbb{M}({\mathfrak{\mathrm{\alpha }}}-1)} \int _{0}^{{\mathrm{b}}} \mathrm{g}\bigl(\xi, \mathrm{u}(\xi ), {}_{0}^{{ \mathcal{ABC}}}\mathcal{D}^{\mathfrak{\mathrm{\alpha }}}_{\xi } \mathrm{u} (\xi )\bigr) \,d\xi \\ &{} - \frac{\mathrm{t}({\mathfrak{\mathrm{\alpha }}}-1)}{{\mathrm{b}}\mathbb{M}({\mathfrak{\mathrm{\alpha }}}-1) \Gamma ({\mathfrak{\mathrm{\alpha }}})} \int _{0}^{{\mathrm{b}}} \mathrm{g}\bigl(\xi, \mathrm{u}(\xi ),{}_{0}^{{\mathcal{ABC}}}\mathcal{D}^{ \mathfrak{\mathrm{\alpha }}}_{\xi } \mathrm{u}(\xi )\bigr) ({\mathrm{b}}- \xi )^{\mathfrak{\mathfrak{\mathrm{\alpha }}-1}}\,d\xi \\ &{} + \frac{2-{\mathfrak{\mathrm{\alpha }}}}{\mathbb{M}({\mathfrak{\mathrm{\alpha }}}-1)} \int _{0}^{\mathrm{t}} \mathrm{g}\bigl(\xi, \mathrm{u} (\xi ),{}_{0}^{{ \mathcal{ABC}}}\mathcal{D}^{\mathfrak{\mathrm{\alpha }}}_{\xi } \mathrm{u}(\xi )\bigr) \,d\xi \\ & {}+ \frac{{\mathfrak{\mathrm{\alpha }}}-1}{\mathbb{M}({\mathfrak{\mathrm{\alpha }}}-1)\Gamma ({\mathfrak{\mathrm{\alpha }}})} \int _{0}^{\mathrm{t}} \mathrm{g}\bigl(\xi, \mathrm{u}(\xi ),{}_{0}^{{ \mathcal{ABC}}}\mathcal{D}^{\mathfrak{\mathrm{\alpha }}}_{\xi } \mathrm{u}(\xi )\bigr) (\mathrm{t} - \xi )^{{\mathfrak{\mathrm{\alpha }}}-1} \,d\xi. \end{aligned}$$

For the proof of our main results, we employ the following hypotheses:

$( \mathrm{A}_{1})$ There exists a constant $\mathcal{L}_{\mathrm{g}} >0 $ such that, for any $\mathrm{u}, \overline{\mathrm{u}}, \mathrm{v}, \overline{\mathrm{v}} \in \mathbb{X} $, one has

$$\begin{aligned} \bigl\vert \mathrm{g}(\mathrm{t}, \mathrm{u}, \mathrm{v})-\mathrm{g}( \mathrm{t}, \overline{\mathrm{u}}, \overline{\mathrm{v}}) \bigr\vert \leq \mathcal{L}_{\mathrm{g}} \bigl( \vert \mathrm{u}-\overline{\mathrm{u}} \vert + \vert \mathrm{v}-\overline{\mathrm{v}} \vert \bigr). \end{aligned}$$

$( \mathrm{A}_{2}) $ There exist constants $\mathbf{A}_{\mathrm{g}}, \mathbf{B}_{\mathrm{g}}, \mathbf{C}_{ \mathrm{g}}>0$ such that

$$\begin{aligned} \bigl\vert \mathrm{g}(\mathrm{t}, \mathrm{u}, \mathrm{v}) \bigr\vert \leq \mathbf{A}_{ \mathrm{g}} + \mathbf{B}_{\mathrm{g}} \vert \mathrm{u} \vert + \mathbf{C}_{ \mathrm{g}} \vert \mathrm{v} \vert . \end{aligned}$$

### Theorem 2

*Under assumption* $( \mathrm{A}_{1})$, *BVP* (3) *has the unique solution if*

9 $$\begin{aligned} 4 \biggl(\mathcal{L}_{\mathrm{g}} + \frac{\mathcal{L}_{\mathrm{g}} ^{2}}{1-\mathcal{L}_{\mathrm{g}} } \biggr) \frac{{\mathrm{b}}\Gamma ({\mathfrak{\mathrm{\alpha }}}+1)+ {\mathrm{b}}^{\mathfrak{\mathrm{\alpha }}}}{\mathbb{M}({\mathfrak{\mathrm{\alpha }}}-1)\Gamma ({\mathfrak{\mathrm{\alpha }}}+1)}< 1. \end{aligned}$$

### Proof

Let $\mathcal{H}: \mathbb{X}\rightarrow \mathbb{X} $ be defined by

$$\begin{aligned} \mathcal{H}\mathrm{u}(\mathrm{t}) ={}& \frac{\mathrm{t}\mathrm{u}_{1} + \mathrm{u}_{0}({\mathrm{b}}-\mathrm{t})}{{\mathrm{b}}} - \frac{\mathrm{t}(2-{\mathfrak{\mathrm{\alpha }}})}{{\mathrm{b}} \mathbb{M}({\mathfrak{\mathrm{\alpha }}}-1)} \int _{0}^{{\mathrm{b}}} \mathrm{g}\bigl(\xi, \mathrm{u}(\xi ),{}_{0}^{{ \mathcal{ABC}}}\mathcal{D}^{\mathfrak{\mathrm{\alpha }}}_{\xi } \mathrm{u}(\xi )\bigr) \,d\xi \\ &{} - \frac{\mathrm{t}({\mathfrak{\mathrm{\alpha }}}-1)}{{\mathrm{b}}\mathbb{M}({\mathfrak{\mathrm{\alpha }}}-1)\Gamma ({\mathfrak{\mathrm{\alpha }}})} \int _{0}^{{\mathrm{b}}}\mathrm{g}\bigl(\xi, \mathrm{u}(\xi ),{}_{0}^{{ \mathcal{ABC}}}\mathcal{D}^{\mathfrak{\mathrm{\alpha }}}_{\xi } \mathrm{u}(\xi )\bigr) ({\mathrm{b}}-\xi )^{{\mathfrak{\mathrm{\alpha }}}-1}\,d \xi \\ & {}+ \frac{2-{\mathfrak{\mathrm{\alpha }}}}{\mathbb{M}({\mathfrak{\mathrm{\alpha }}}-1)} \int _{0}^{\mathrm{t}} \mathrm{g}\bigl(\xi, \mathrm{u}(\xi ),{}_{0}^{{ \mathcal{ABC}}}\mathcal{D}^{\mathfrak{\mathrm{\alpha }}}_{\xi } \mathrm{u}(\xi )\bigr) \,d\xi \\ &{}+ \frac{{\mathfrak{\mathrm{\alpha }}}-1}{\mathbb{M}({\mathfrak{\mathrm{\alpha }}}-1)\Gamma ({\mathfrak{\mathrm{\alpha }}})} \int _{0}^{\mathrm{t}} \mathrm{g}\bigl(\xi, \mathrm{u}(\xi ),{}_{0}^{{ \mathcal{ABC}}}\mathcal{D}^{\mathfrak{\mathrm{\alpha }}}_{\xi } \mathrm{u}(\xi )\bigr) (\mathrm{t}-\xi )^{{\mathfrak{\mathrm{\alpha }}}-1}\,d \xi. \end{aligned}$$

To examine the uniqueness of the solution of the problem, firstly we show that $\mathcal{H}$ is a contraction operator. To this end, suppose $\mathrm{u}, \overline{\mathrm{u}} \in \mathbb{ X}$, then

10 $$\begin{aligned} & \Vert \mathcal{H} \mathrm{u}-\mathcal{H} \overline{ \mathrm{u}} \Vert \\ & \quad= \max_{ \mathrm{t} \in \mathcal{J}} \biggl\vert \frac{\mathrm{t}(2-{\mathfrak{\mathrm{\alpha }}})}{{\mathrm{b}} \mathbb{M}({\mathfrak{\mathrm{\alpha }}}-1)} \int _{0}^{{\mathrm{b}}} \bigl[ \mathrm{g}\bigl(\xi, \mathrm{u}(\xi ),{}_{0}^{{ \mathcal{ABC}}}\mathcal{D}^{\mathfrak{\mathrm{\alpha }}}_{\xi } \mathrm{u}(\xi )\bigr)- \mathrm{g}\bigl(\xi, \overline{\mathrm{u}}(\xi ),{}_{0}^{{ \mathcal{ABC}}}\mathcal{D}^{\mathfrak{\mathrm{\alpha }}}_{\xi } \overline{\mathrm{u}}(\xi )\bigr) \bigr] \,d\xi \\ &\qquad{} + \frac{\mathrm{t}({\mathfrak{\mathrm{\alpha }}}-1)}{{\mathrm{b}}\mathbb{M}({\mathfrak{\mathrm{\alpha }}}-1) \Gamma ({\mathfrak{\mathrm{\alpha }}})} \int _{0}^{{\mathrm{b}}} \bigl[ \mathrm{g}\bigl(\xi, \mathrm{u}(\xi ),{}_{0}^{{\mathcal{ABC}}}\mathcal{D}^{ \mathfrak{\mathrm{\alpha }}}_{\xi } \mathrm{u}(\xi )\bigr)- \mathrm{g}\bigl(\xi, \overline{\mathrm{u}}(\xi ),{}_{0}^{{\mathcal{ABC}}}\mathcal{D}^{ \mathfrak{\mathrm{\alpha }}}_{\xi } \overline{\mathrm{u}}(\xi )\bigr) \bigr] \\ &\qquad{}\times({ \mathrm{b}}-\xi )^{{\mathfrak{\mathrm{\alpha }}}-1} \,d\xi \\ &\qquad{} + \frac{2-{\mathfrak{\mathrm{\alpha }}}}{\mathbb{M}({\mathfrak{\mathrm{\alpha }}}-1)} \int _{0}^{{\mathrm{b}}} \bigl[ \mathrm{g}\bigl(\xi, \mathrm{u}(\xi ),{}_{0}^{{ \mathcal{ABC}}}\mathcal{D}^{\mathfrak{\mathrm{\alpha }}}_{\xi } \mathrm{u}(\xi )\bigr)- \mathrm{g}\bigl(\xi, \overline{\mathrm{u}}(\xi ),{}_{0}^{{ \mathcal{ABC}}}\mathcal{D}^{\mathfrak{\mathrm{\alpha }}}_{\xi } \overline{\mathrm{u}}(\xi )\bigr) \bigr] \,d\xi \\ &\qquad{} + \frac{{\mathfrak{\mathrm{\alpha }}}-1}{\mathbb{M}({\mathfrak{\mathrm{\alpha }}}-1)\Gamma ({\mathfrak{\mathrm{\alpha }}})} \int _{0}^{{\mathrm{b}}} \bigl[ \mathrm{g}\bigl(\xi, \mathrm{u}(\xi ),{}_{0}^{{ \mathcal{ABC}}}\mathcal{D}^{\mathfrak{\mathrm{\alpha }}}_{\xi } \mathrm{u}(\xi )\bigr)- \mathrm{g}\bigl(\xi, \overline{\mathrm{u}}(\xi ),{}_{0}^{{ \mathcal{ABC}}}\mathcal{D}^{\mathfrak{\mathrm{\alpha }}}_{\xi } \overline{\mathrm{u}}(\xi )\bigr) \bigr] \\ &\qquad{}\times (\mathrm{t} - \xi )^{{ \mathfrak{\mathrm{\alpha }}}-1} \,d\xi \biggr\vert \\ & \quad\leq \max_{\mathrm{t} \in \mathcal{J}} \biggl[ \frac{ \vert \mathrm{t} \vert (2-{\mathfrak{\mathrm{\alpha }}})}{{\mathrm{b}} \mathbb{M}({\mathfrak{\mathrm{\alpha }}}-1)} \int _{0}^{{\mathrm{b}}} \mathcal{L}_{\mathrm{g}} \bigl( \bigl\vert \mathrm{u}(\xi )- \overline{\mathrm{u}} (\xi ) \bigr\vert + \bigl\vert _{0}^{{ \mathcal{ABC}}}\mathcal{D}^{\mathfrak{\mathrm{\alpha }}}_{\xi } \mathrm{u}(\xi )- {}_{0}^{{\mathcal{ABC}}}\mathcal{D}^{ \mathfrak{\mathrm{\alpha }}}_{\xi } \overline{\mathrm{u}}(\xi ) \bigr\vert \bigr)\,d\xi \\ &\qquad{}+\frac{ \vert \mathrm{t} \vert ({\mathfrak{\mathrm{\alpha }}}-1)}{{\mathrm{b}}\mathbb{M}({\mathfrak{\mathrm{\alpha }}}-1)\Gamma ({\mathfrak{\mathrm{\alpha }}})} \int _{0}^{{\mathrm{b}}} \mathcal{L}_{\mathrm{g}} \bigl( \bigl\vert \mathrm{u}(\xi )- \overline{\mathrm{u}}(\xi ) \bigr\vert + \bigl\vert {}_{0}^{{ \mathcal{ABC}}}\mathcal{D}^{\mathfrak{\mathrm{\alpha }}}_{\xi } \mathrm{u}(\xi )- {}_{0}^{{\mathcal{ABC}}}\mathcal{D}^{ \mathfrak{\mathrm{\alpha }}}_{\xi } \overline{\mathrm{u}}(\xi ) \bigr\vert \bigr) \\ &\qquad{}\times({\mathrm{b}}-\xi )^{{\mathfrak{\mathrm{\alpha }}}-1} \,d\xi \\ &\qquad{}+ \frac{2-{\mathfrak{\mathrm{\alpha }}}}{\mathbb{M}({\mathfrak{\mathrm{\alpha }}}-1)} \int _{0}^{{\mathrm{b}}} \mathcal{L}_{\mathrm{g}} \bigl( \bigl\vert \mathrm{u}(\xi )- \overline{\mathrm{u}}(\xi ) \bigr\vert + \bigl\vert {}_{0}^{{ \mathcal{ABC}}}\mathcal{D}^{\mathfrak{\mathrm{\alpha }}}_{\xi } \mathrm{u}(\xi )- {}_{0}^{{\mathcal{ABC}}}\mathcal{D}^{ \mathfrak{\mathrm{\alpha }}}_{\xi } \overline{\mathrm{u} }(\xi ) \bigr\vert \bigr)\,d\xi \\ &\qquad{} +\frac{{\mathfrak{\mathrm{\alpha }}}-1}{\mathbb{M}({\mathfrak{\mathrm{\alpha }}}-1)\Gamma ({\mathfrak{\mathrm{\alpha }}})} \int _{0}^{{\mathrm{b}}} \mathcal{L}_{\mathrm{g}} \bigl( \bigl\vert \mathrm{u}(\xi )- \overline{\mathrm{u}}(\xi ) \bigr\vert + \bigl\vert {}_{0}^{{ \mathcal{ABC}}}\mathcal{D}^{\mathfrak{\mathrm{\alpha }}}_{\xi } \mathrm{u}(\xi ) - {}_{0}^{{\mathcal{ABC}}}\mathcal{D}^{ \mathfrak{\mathrm{\alpha }}}_{\xi } \overline{\mathrm{u}}(\xi ) \bigr\vert \bigr)\,d\xi \biggr]. \end{aligned}$$

Now, we have

$$\begin{aligned} &\bigl\vert {}_{0}^{{\mathcal{{\mathcal{ABC}}}}}\mathcal{D}^{ \mathfrak{\mathrm{\alpha }}}_{\xi } \mathrm{u}(\mathrm{t}) - {}_{0}^{{ \mathcal{ABC}}}\mathcal{D}^{\mathfrak{\mathrm{\alpha }}}_{\xi } \overline{\mathrm{u}}(\mathrm{t}) \bigr\vert \\ &\quad = \bigl\vert \mathrm{g}\bigl( \mathrm{t}, \mathrm{u}(\mathrm{t}),{}_{0}^{{\mathcal{ABC}}} \mathcal{D}^{\mathfrak{\mathrm{\alpha }}}_{\xi } \mathrm{u}(\mathrm{t})\bigr) - \mathrm{g} \bigl(\mathrm{t}, \overline{\mathrm{u}}(\mathrm{t}), {}_{0}^{{ \mathcal{ABC}}} \mathcal{D}^{\mathfrak{\mathrm{\alpha }}}_{\xi } \overline{\mathrm{u}}(\mathrm{t})\bigr) \bigr\vert \\ &\quad\leq \mathcal{L}_{\mathrm{g}} \bigl( \bigl\vert \mathrm{u}(\mathrm{t}) - \overline{\mathrm{u}}(\mathrm{t}) \bigr\vert + \bigl\vert {}_{0}^{{ \mathcal{{\mathcal{ABC}}}}} \mathcal{D}^{\mathfrak{\mathrm{\alpha }}}_{ \xi } \mathrm{u}(\mathrm{t}) - _{0}^{{\mathcal{{\mathcal{ABC}}}}} \mathcal{D}^{\mathfrak{\mathrm{\alpha }}}_{\xi }\overline{ \mathrm{u} }( \mathrm{t}) \bigr\vert \bigr). \end{aligned}$$

This implies

11 $$\begin{aligned} \bigl\vert {}_{0}^{{\mathcal{{\mathcal{ABC}}}}}\mathcal{D}^{ \mathfrak{\mathrm{\alpha }}}_{\xi } \mathrm{u}(\xi )- {}_{0}^{{ \mathcal{ABC}}}\mathcal{D}^{\mathfrak{\mathrm{\alpha }}}_{\xi } \overline{\mathrm{u}}(\mathrm{t}) \bigr\vert \leq \biggl( \frac{\mathcal{L}_{\mathrm{g}} }{1-\mathcal{L}_{\mathrm{g}} } \biggr) \bigl\vert \mathrm{u}(\mathrm{t})- \overline{\mathrm{u}}(\mathrm{t}) \bigr\vert . \end{aligned}$$

Using equation (11) in equation (10), we get

$$\begin{aligned} \Vert \mathcal{H} \mathrm{u}-\mathcal{H} \overline{\mathrm{u}} \Vert \leq 4 \biggl(\mathcal{L}_{\mathrm{g}} + \frac{\mathcal{L}_{\mathrm{g}} ^{2}}{1-\mathcal{L}_{\mathrm{g}} } \biggr) \frac{{\mathrm{b}}\Gamma ({\mathfrak{\mathrm{\alpha }}}+1)+{\mathrm{b}}^{\mathfrak{\mathrm{\alpha }}}}{\mathbb{M} ({\mathfrak{\mathrm{\alpha }}}-1)\Gamma ({\mathfrak{\mathrm{\alpha }}}+1)} \Vert \mathrm{u}-\overline{\mathrm{u}} \Vert . \end{aligned}$$

In view of equation (9), our BVP (3) has the unique solution. Hence $\mathcal{H} $ is a contraction operator. Hence $\mathcal{H} $ is defined under consideration of Krasnoselskii’s fixed point theorem as follows:

$$\begin{aligned} \mathcal{H}\mathrm{u}(\mathrm{t}) = \mathcal{F}\mathrm{u}(\mathrm{t})+ \mathcal{N}\mathrm{u}(\mathrm{t}), \end{aligned}$$

where

$$\begin{aligned} \mathcal{F}\mathrm{u}(\mathrm{t}) ={}& \frac{\mathrm{t}\mathrm{u}_{1} + \mathrm{u}_{0}({\mathrm{b}}-\mathrm{t})}{{\mathrm{b}}} - \frac{\mathrm{t}(2-{\mathfrak{\mathrm{\alpha }}})}{{\mathrm{b}} \mathbb{M}({\mathfrak{\mathrm{\alpha }}}-1)} \int _{0}^{{\mathrm{b}}} \mathrm{g}\bigl(\xi, \mathrm{u}(\xi ),{}_{0}^{{ \mathcal{ABC}}}\mathcal{D}^{\mathfrak{\mathrm{\alpha }}}_{\xi } \mathrm{u}(\xi )\bigr) \,d\xi \\ & {}- \frac{\mathrm{t}({\mathfrak{\mathrm{\alpha }}}-1)}{{\mathrm{b}}\mathbb{M}({\mathfrak{\mathrm{\alpha }}}-1)\Gamma ({\mathfrak{\mathrm{\alpha }}})} \int _{0}^{{\mathrm{b}}}\mathrm{g}\bigl(\xi, \mathrm{u}(\xi ),{}_{0}^{{ \mathcal{ABC}}}\mathcal{D}^{\mathfrak{\mathrm{\alpha }}}_{\xi } \mathrm{u}(\xi )\bigr) (\mathfrak{\mathrm{b}}-\xi )^{{ \mathfrak{\mathrm{\alpha }}}-1}\,d\xi, \\ \mathcal{N}\mathrm{u}(\mathrm{t}) ={}& + \frac{2-{\mathfrak{\mathrm{\alpha }}}}{\mathbb{M}({\mathfrak{\mathrm{\alpha }}}-1)} \int _{0}^{\mathrm{t}} \mathrm{g}\bigl(\xi, \mathrm{u}(\xi ),{}_{0}^{{ \mathcal{ABC}}}\mathcal{D}^{\mathfrak{\mathrm{\alpha }}}_{\xi } \mathrm{u}(\xi )\bigr) \,d\xi \\ &{} + \frac{{\mathfrak{\mathrm{\alpha }}}-1}{\mathbb{M}({\mathfrak{\mathrm{\alpha }}}-1)\Gamma ({\mathfrak{\mathrm{\alpha }}})} \int _{0}^{\mathrm{t}} \mathrm{g}\bigl(\xi, \mathrm{u}(\xi ),{}_{0}^{{ \mathcal{ABC}}}\mathcal{D}^{\mathfrak{\mathrm{\alpha }}}_{\xi } \mathrm{u}(\xi )\bigr) (\mathrm{t}-\xi )^{{\mathfrak{\mathrm{\alpha }}}-1}\,d \xi. \end{aligned}$$

 □

### Theorem 3

*Under the contemplation of assumptions* $( \mathrm{A}_{1}) $ *and* $(\mathrm{A}_{2})$, *our BVP has at least one solution with the condition*

12 $$\begin{aligned} 2 \biggl(\mathcal{L}_{\mathrm{g}} + \frac{\mathcal{L}_{\mathrm{g}} ^{2}}{1-\mathcal{L}_{\mathrm{g}} } \biggr) \frac{\mathfrak{\mathrm{b}}\Gamma ({\mathfrak{\mathrm{\alpha }}}+1)+\mathfrak{\mathrm{b}}^{\mathfrak{\mathrm{\alpha }}}}{\mathbb{M}({\mathfrak{\mathrm{\alpha }}}-1)\Gamma ({\mathfrak{\mathrm{\alpha }}}+1)}< 1. \end{aligned}$$

### Proof

Let $\mathcal{W} = \{ \mathrm{u} \in \mathbb{X}: \|\mathrm{u}\| \leq \mathrm{r} \} $ be a closed bounded set and $\mathrm{u}, \overline{\mathrm{u}} \in \mathcal{W}$. Now

13 $$\begin{aligned} &\Vert \mathcal{F} \mathrm{u} - \mathcal{F} \overline{\mathrm{u}} \Vert \\ &\quad = \max_{\mathrm{t} \in \mathcal{J}} \bigl\vert \mathcal{F} \mathrm{u}( \mathrm{t}) - \mathcal{F} \overline{\mathrm{u}}(\mathrm{t}) \bigr\vert \\ &\quad =\max_{\mathrm{t} \in \mathcal{J}} \frac{ \vert \mathrm{t}(2-{\mathfrak{\mathrm{\alpha }}}) \vert }{\mathfrak{\mathrm{b}} \mathbb{M}({\mathfrak{\mathrm{\alpha }}}-1)} \int _{0}^{\mathfrak{\mathrm{b}}} \bigl\vert \mathrm{g}\bigl(\xi, \mathrm{u}(\xi ), {}_{0}^{{\mathcal{ABC}}}\mathcal{D}^{\mathfrak{\mathrm{\alpha }}}_{\xi } \mathrm{u}(\xi )\bigr)- \mathrm{g}\bigl(\xi,\overline{\mathrm{u}}(\xi ), _{0}^{{ \mathcal{ABC}}}\mathcal{D}^{\mathfrak{\mathrm{\alpha }}}_{\xi } \overline{ \mathrm{u}}(\xi )\bigr) \bigr\vert \,d\xi \\ &\qquad{}+\max_{\mathrm{t} \in \mathcal{J}} \frac{ \vert \mathrm{t} \vert ({\mathfrak{\mathrm{\alpha }}}-1)}{\mathfrak{\mathrm{b}}\mathbb{M}({\mathfrak{\mathrm{\alpha }}}-1)\Gamma ({\mathfrak{\mathrm{\alpha }}})} \int _{0}^{\mathfrak{\mathrm{b}}} \bigl\vert \mathrm{g}\bigl(\xi, \mathrm{u}(\xi ), {}_{0}^{{\mathcal{ABC}}}\mathcal{D}^{\mathfrak{\mathrm{\alpha }}}_{\xi } \mathrm{u}(\xi )\bigr) - \mathrm{g}\bigl(\xi, \overline{\mathrm{u}}(\xi ),{}_{0}^{{ \mathcal{ABC}}}\mathcal{D}^{\mathfrak{\mathrm{\alpha }}}_{\xi } \overline{\mathrm{u}}(\xi )\bigr) \bigr\vert \\ &\qquad{}\times(\mathfrak{\mathrm{b}}-\xi )^{{ \mathfrak{\mathrm{\alpha }}}-1} \,d\xi \\ &\quad\leq \max_{\mathrm{t} \in \mathcal{J}} \frac{ \vert \mathrm{t} \vert (2-{\mathfrak{\mathrm{\alpha }}})}{\mathfrak{\mathrm{b}} \mathbb{M}({\mathfrak{\mathrm{\alpha }}}-1)} \int _{0}^{\mathfrak{\mathrm{b}}} \mathcal{L}_{\mathrm{g}} \bigl( \bigl\vert \mathrm{u}(\xi ) - \overline{\mathrm{u}}(\xi ) \bigr\vert + \bigl\vert {}_{0}^{{\mathcal{ABC}}}\mathcal{D}^{\mathfrak{\mathrm{\alpha }}}_{ \xi } \mathrm{u}(\xi ) - {}_{0}^{{\mathcal{ABC}}}\mathcal{D}^{ \mathfrak{\mathrm{\alpha }}}_{\xi } \overline{\mathrm{u}}(\xi ) \bigr\vert \bigr)\,d\xi \\ &\qquad{}+\max_{\mathrm{t} \in \mathcal{J}} \frac{ \vert \mathrm{t} \vert ({\mathfrak{\mathrm{\alpha }}}-1)}{\mathfrak{\mathrm{b}}\mathbb{M}({\mathfrak{\mathrm{\alpha }}}-1) \Gamma ({\mathfrak{\mathrm{\alpha }}})} \int _{0}^{ \mathfrak{\mathrm{b}}} \mathcal{L}_{\mathrm{g}} \bigl( \bigl\vert \mathrm{u}(\xi )- \overline{\mathrm{u}}(\xi ) \bigr\vert + \bigl\vert {}_{0}^{{ \mathcal{ABC}}}\mathcal{D}^{\mathfrak{\mathrm{\alpha }}}_{\xi } \mathrm{u}(\xi ) - {}_{0}^{{\mathcal{ABC}}}\mathcal{D}^{ \mathfrak{\mathrm{\alpha }}}_{\xi } \overline{\mathrm{u}}(\xi ) \bigr\vert \bigr) \\ &\qquad{}\times (\mathfrak{\mathrm{b}}-\xi )^{{\mathfrak{\mathrm{\alpha }}}-1} \,d\xi \\ &\quad\leq 2 \biggl(\mathcal{L}_{\mathrm{g}}+ \frac{\mathcal{L}_{\mathrm{g}}^{2}}{1-\mathcal{L}_{\mathrm{g}}} \biggr) \frac{\mathfrak{\mathrm{b}}\Gamma ({\mathfrak{\mathrm{\alpha }}}+1) + \mathfrak{\mathrm{b}}^{\mathfrak{\mathrm{\alpha }}}}{\mathbb{M}(\mathfrak{\mathrm{\alpha }}-1) \Gamma ({\mathfrak{\mathrm{\alpha }}}+1)} \Vert \mathrm{u}- \overline{\mathrm{u}} \Vert . \end{aligned}$$

In view of inequality (12), $\mathcal{F} $ is a contraction mapping. The next step is to prove the continuity and compactness for $\mathcal{N} $. We show that our operator $\mathcal{N} $ is bounded. To examine the equicontinuity for the operator, one has

14 $$\begin{aligned} \begin{aligned} \Vert \mathcal{N}\mathrm{u} \Vert = {}&\max _{\mathrm{t} \in \mathcal{J}} \bigl\vert \mathcal{N}\mathrm{u}(\mathrm{t}) \bigr\vert \\ ={}& \max_{\mathrm{t} \in \mathcal{J}} \biggl\vert \frac{2-{\mathfrak{\mathrm{\alpha }}}}{\mathbb{M}({\mathfrak{\mathrm{\alpha }}}-1)} \int _{0}^{\mathrm{t}} \mathrm{g}\bigl(\xi, \mathrm{u}(\xi ),{}_{0}^{{ \mathcal{ABC}}}\mathcal{D}^{\mathfrak{\mathrm{\alpha }}}_{\xi } \mathrm{u}(\xi )\bigr) \,d\xi \\ &{} + \frac{{\mathfrak{\mathrm{\alpha }}}-1}{\mathbb{M}({\mathfrak{\mathrm{\alpha }}}-1)\Gamma ({\mathfrak{\mathrm{\alpha }}})} \int _{0}^{\mathrm{t}} \mathrm{g}\bigl(\xi, \mathrm{u}(\xi ),{}_{0}^{{ \mathcal{ABC}}}\mathcal{D}^{\mathfrak{\mathrm{\alpha }}}_{\xi } \mathrm{u}(\xi )\bigr) (\mathrm{t}-\xi )^{{\mathfrak{\mathrm{\alpha }}}-1}\,d \xi \biggr\vert \\ \leq {}&\frac{2-{\mathfrak{\mathrm{\alpha }}}}{\mathbb{M}({\mathfrak{\mathrm{\alpha }}}-1)} \int _{0}^{\mathrm{t}} \bigl[ \mathbf{A}_{\mathrm{g}} + \mathbf{B}_{ \mathrm{g}} \bigl\vert \mathrm{u}(\xi ) \bigr\vert + \mathbf{C}_{\mathrm{g}} \bigl\vert {}_{0}^{{ \mathcal{ABC}}} \mathcal{D}^{\mathfrak{\mathrm{\alpha }}}_{\mathrm{t}} \bigl( \mathrm{u}(\xi )\bigr) \bigr\vert \bigr] \,d\xi \\ &{} + \frac{{\mathfrak{\mathrm{\alpha }}}-1}{\mathbb{M}({\mathfrak{\mathrm{\alpha }}}-1)\Gamma ({\mathfrak{\mathrm{\alpha }}})} \int _{0}^{\mathrm{t}} \bigl[ \mathbf{A}_{\mathrm{g}} + \mathbf{B}_{ \mathrm{g}} \bigl\vert \mathrm{u}(\xi ) \bigr\vert + \mathbf{C}_{\mathrm{g}} \bigl\vert {}_{0}^{{ \mathcal{ABC}}} \mathcal{D}^{\mathfrak{\mathrm{\alpha }}}_{\xi } \bigl( \mathrm{u}(\xi )\bigr) \bigr\vert \bigr] (\mathrm{t}-\xi )^{{ \mathfrak{\mathrm{\alpha }}}-1}\,d\xi. \end{aligned} \end{aligned}$$

Now

$$\begin{aligned} \bigl\vert {}_{0}^{{\mathcal{ABC}}}\mathcal{D}^{\mathfrak{\mathrm{\alpha }}}_{ \mathrm{t}} \bigl(\mathrm{u}(\mathrm{t})\bigr) \bigr\vert & = \bigl\vert \mathrm{g}\bigl( \mathrm{t}, \mathrm{u}(\mathrm{t}),{}_{0}^{{\mathcal{ABC}}} \mathcal{D}^{\mathfrak{\mathrm{\alpha }}}_{\mathrm{t}}\mathrm{u}( \mathrm{t})\bigr) \bigr\vert \\ & \leq \mathbf{A}_{\mathrm{g}} + \mathbf{B}_{\mathrm{g}} \bigl\vert \mathrm{u}( \mathrm{t}) \bigr\vert + \mathbf{C}_{\mathrm{g}} \bigl\vert _{0}^{{\mathcal{ABC}}} \mathcal{D}^{\mathfrak{\mathrm{\alpha }}}_{\mathrm{t}} \bigl( \mathrm{u}( \mathrm{t})\bigr) \bigr\vert . \end{aligned}$$

This implies

15 $$\begin{aligned} \bigl\vert {}_{0}^{{\mathcal{ABC}}}\mathcal{D}^{\mathfrak{\mathrm{\alpha }}}_{ \mathrm{t}} \bigl(\mathrm{u}(\mathrm{t})\bigr) \bigr\vert \leq \frac{\mathbf{A}_{\mathrm{g}}}{1-\mathbf{C}_{\mathrm{g}}} + \frac{\mathbf{B}_{\mathrm{g}}}{1-\mathbf{C}_{\mathrm{g}}} \bigl\vert \mathrm{u}( \mathrm{t}) \bigr\vert . \end{aligned}$$

Using equation (15) in equation (14), we get

$$\begin{aligned} \Vert \mathcal{N}\mathrm{u} \Vert \leq \frac{\mathfrak{\mathrm{b}}\Gamma ({\mathfrak{\mathrm{\alpha }}} + 1)+ \mathfrak{\mathrm{b}}^{\mathfrak{\mathrm{\alpha }}}}{\mathbb{M}({\mathfrak{\mathrm{\alpha }}}-1)\Gamma ({\mathfrak{\mathrm{\alpha }}}+1)} \biggl[ \mathbf{A}_{\mathrm{g}} + \frac{\mathbf{A}_{\mathrm{g}}.\mathbf{C}_{\mathrm{g}}}{1-\mathbf{C}_{\mathrm{g}}} + \biggl(\mathbf{B}_{\mathrm{g}}+ \frac{\mathbf{B}_{\mathrm{g}}}{1-\mathbf{C}_{\mathrm{g}}} \biggr) \mathfrak{M } \biggr]. \end{aligned}$$

Hence, $\mathcal{N} $ is bounded. Now to prove equicontinuity for $\mathcal{N} $, let $\mathrm{t}_{1} < \mathrm{t}_{2}\in \mathcal{J}$, then

16 $$\begin{aligned} &\bigl\vert \mathcal{N}\mathrm{u}( \mathrm{t}_{2}) - \mathcal{N}\mathrm{u}( \mathrm{t}_{1}) \bigr\vert \\ &\quad\leq \frac{2-{\mathfrak{\mathrm{\alpha }}}}{\mathbb{M}({\mathfrak{\mathrm{\alpha }}}-1)} \biggl[ \int _{\mathrm{t}_{1}}^{\mathrm{t}_{2}} \bigl\vert \mathrm{g}\bigl(\xi, \mathrm{u}(\xi ),{}_{0}^{{\mathcal{ABC}}}\mathcal{D}^{ \mathfrak{\mathrm{\alpha }}}_{\xi } \mathrm{u}(\xi )\bigr) \bigr\vert \,d\xi \biggr] \\ &\qquad{} + \frac{{\mathfrak{\mathrm{\alpha }}}-1}{\mathbb{M}({\mathfrak{\mathrm{\alpha }}}-1)\Gamma ({\mathfrak{\mathrm{\alpha }}})} \int _{0}^{\mathrm{t}_{1}}\bigl[(\mathrm{t}_{1}-\xi )^{{ \mathfrak{\mathrm{\alpha }}}-1}-(\mathrm{t}_{2}-\xi )^{{ \mathfrak{\mathrm{\alpha }}}-1}\bigr] \bigl\vert \mathrm{g}\bigl(\xi, \mathrm{u}(\xi ), {}_{0}^{{ \mathcal{ABC}}} \mathcal{D}^{\mathfrak{\mathrm{\alpha }}}_{\xi } \mathrm{u}(\xi )\bigr) \bigr\vert \,d \xi \\ &\qquad{}+\frac{{\mathfrak{\mathrm{\alpha }}}-1}{\mathbb{M}({\mathfrak{\mathrm{\alpha }}}-1)\Gamma ({\mathfrak{\mathrm{\alpha }}})} \int _{\mathrm{t}_{1}}^{\mathrm{t}_{2}}(\mathrm{t}_{2}-\xi )^{{ \mathfrak{\mathrm{\alpha }}}-1} \bigl\vert \mathrm{g}\bigl(\xi, \mathrm{u}(\xi ), _{0}^{{ \mathcal{ABC}}}\mathcal{D}^{\mathfrak{\mathrm{\alpha }}}_{\xi } \mathrm{u}(\xi )\bigr) \bigr\vert \,d\xi \\ &\quad \leq \frac{2-{\mathfrak{\mathrm{\alpha }}}}{\mathbb{M}({\mathfrak{\mathrm{\alpha }}}-1)} \int _{\mathrm{t}_{1}}^{\mathrm{t}_{2}} \bigl[ \mathbf{A}_{\mathrm{g}} + \mathbf{B}_{\mathrm{g}} \bigl\vert \mathrm{u}(\xi ) \bigr\vert + \mathbf{C}_{\mathrm{g}} \bigl\vert {}_{0}^{{ \mathcal{ABC}}} \mathcal{D}^{\mathfrak{\mathrm{\alpha }}}_{\xi } \mathrm{u}(\xi ) \bigr\vert \bigr]\,d \xi \\ &\qquad{} + \frac{{\mathfrak{\mathrm{\alpha }}}-1}{\mathbb{M}({\mathfrak{\mathrm{\alpha }}}-1)\Gamma ({\mathfrak{\mathrm{\alpha }}})} \int _{0}^{\mathrm{t}_{1}}\bigl[(\mathrm{t}_{1}-\xi )^{{ \mathfrak{\mathrm{\alpha }}}-1}-(\mathrm{t}_{2}-\xi )^{{ \mathfrak{\mathrm{\alpha }}}-1}\bigr] \\ &\qquad{}\times\bigl[ \mathbf{A}_{\mathrm{g}} + \mathbf{B}_{\mathrm{g}} \bigl\vert \mathrm{u}(\xi ) \bigr\vert + \mathbf{C}_{\mathrm{g}} \bigl\vert {}_{0}^{{ \mathcal{ABC}}} \mathcal{D}^{\mathfrak{\mathrm{\alpha }}}_{\xi } \mathrm{u}(\xi ) \bigr\vert \bigr]\,d \xi \\ &\qquad{}+\frac{{\mathfrak{\mathrm{\alpha }}}-1}{\mathbb{M}({\mathfrak{\mathrm{\alpha }}}-1)\Gamma ({\mathfrak{\mathrm{\alpha }}})} \int _{\mathrm{t}_{1}}^{\mathrm{t}_{2}}(\mathrm{t}_{2}-\xi )^{{ \mathfrak{\mathrm{\alpha }}}-1} \bigl[ \mathbf{A}_{\mathrm{g}} + \mathbf{B}_{\mathrm{g}} \bigl\vert \mathrm{u}(\xi ) \bigr\vert + \mathbf{C}_{\mathrm{g}} \bigl\vert {}_{0}^{{ \mathcal{ABC}}}\mathcal{D}^{\mathfrak{\mathrm{\alpha }}}_{\xi } \mathrm{u}(\xi ) \bigr\vert \bigr]\,d\xi. \end{aligned}$$

Using relation (15) in (16), we get

$$\begin{aligned} &\bigl\vert \mathcal{N}\mathrm{u}(\mathrm{t}_{2}) - \mathcal{N} \mathrm{u}( \mathrm{t}_{1}) \bigr\vert \\ &\quad\leq \frac{(2-\mathrm{\alpha })(\mathrm{t}_{2}-\mathrm{t}_{1})}{\mathbb{M}({\mathfrak{\mathrm{\alpha }}}-1)} \biggl[ \mathbf{A}_{\mathrm{g}} + \frac{\mathbf{A}_{\mathrm{g}}.\mathbf{C}_{\mathrm{g}}}{1-\mathbf{C}_{\mathrm{g}}} + \biggl( \mathbf{B}_{\mathrm{g}}+ \frac{\mathbf{B}_{\mathrm{g}}}{1-\mathbf{C}_{\mathrm{g}}} \biggr) \mathfrak{M } \biggr] \\ &\qquad{}+\frac{(\mathrm{\alpha }-1)(\mathrm{t}_{1}^{\mathfrak{\mathrm{\alpha }}}-\mathrm{t}_{2}^{\mathfrak{\mathrm{\alpha }}}+2(\mathrm{t}_{1}-\mathrm{t}_{2})^{\mathrm{\alpha }})}{\mathbb{M}({\mathfrak{\mathrm{\alpha }}}-1)\Gamma ({\mathfrak{\mathrm{\alpha }}}+1)} \biggl[ \mathbf{A}_{\mathrm{g}} + \frac{\mathbf{A}_{\mathrm{g}}.\mathbf{C}_{\mathrm{g}}}{1-\mathbf{C}_{\mathrm{g}}} + \biggl(\mathbf{B}_{\mathrm{g}}+ \frac{\mathbf{B}_{\mathrm{g}}}{1-\mathbf{C}_{\mathrm{g}}} \biggr) \mathfrak{M } \biggr]. \end{aligned}$$

This shows that $\|\mathcal{N}\mathrm{u}(\mathrm{t}_{2}) - \mathcal{N}\mathrm{u}( \mathrm{t}_{1}) \|\longrightarrow 0 $ as $\mathrm{t}_{2}\longrightarrow \mathrm{t}_{1} $.

Hence, by Arzelá–Ascoli theorem, $\mathcal{N} $ is completely continuous. With all the above, the conditions of Krasnoselskii’s fixed point theorem are fulfilled, and thus the BVP has at least one solution in $\mathcal{W} $. □

## Stability results

Stability analysis has an important impact on the theory of fractional differential equations. In fractional calculus different types of stabilities have been introduced. For our implicit BVP, we use Hyers–Ulam stabilities. In particular, we work on Hyers–Ulam, generalized Hyers–Ulam, Hyers–Ulam–Rassias, and generalized Hyers–Ulam–Rassias stability analysis. For further explanation of these definitions, one can refer to [31–34].

### Definition 4

(Hyers–Ulam stable)

The implicit BVP (3) is Hyers–Ulam stable if there exists a real number $\mathcal{C}_{\mathrm{g}} > 0 $ such that for $\epsilon > 0 $ and for any solution $\mathrm{u} \in \mathbb{X} $ of the inequality

17 $$\begin{aligned} \bigl\vert {}_{0}^{{\mathcal{ABC}}}\mathcal{D}^{\mathfrak{\mathrm{\alpha }}}_{ \mathrm{t}} \mathrm{u}(\mathrm{t}) - \mathrm{g}\bigl(\mathrm{t}, \mathrm{u}(\mathrm{t}), _{0}^{{\mathcal{ABC}}}\mathcal{D}^{ \mathfrak{\mathrm{\alpha }}}_{\mathrm{t}}\mathrm{u}( \mathrm{t})\bigr) \bigr\vert \leq \epsilon, \end{aligned}$$

there is the unique solution $\mathrm{u}^{\star }\in \mathbb{X} $ for our problem (3) such that

$$\begin{aligned} \bigl\Vert \mathrm{u}-\mathrm{u}^{\star } \bigr\Vert \leq \mathcal{C}_{\mathrm{g}} \epsilon. \end{aligned}$$

### Definition 5

(Generalized Hyers–Ulam stable)

Our BVP is generalized Hyers–Ulam stable if there exists $\mathfrak{\varphi } \in \mathcal{C}[0,1] $, $\mathfrak{\varphi }(0)=0 $ such that, for any solution $\mathrm{u} \in \mathbb{X} $ of inequality (17), there is the unique solution $\mathrm{u}^{\star }\in \mathbb{X} $ of problem (3) such that

$$\begin{aligned} \bigl\Vert \mathrm{u}-\mathrm{u}^{\star } \bigr\Vert \leq \mathcal{C}_{\mathrm{g}} \mathfrak{\varphi }(\epsilon ). \end{aligned}$$

### Remark 1

A function $\mathrm{u}\in \mathbb{X} $ is the solution of inequality (17) if there is a function $\Psi \in \mathcal{C}[0, 1] $ that depends on u such that

(1) $|\Psi (\mathrm{t})| \leq \epsilon $;

(2) ${}_{0}^{{\mathcal{ABC}}}\mathcal{D}^{\mathfrak{\mathrm{\alpha }}}_{ \mathrm{t}} \mathrm{u}(\mathrm{t}) = \mathrm{g}(\mathrm{t}, \mathrm{u} (\mathrm{t}), {}_{0}^{{\mathcal{ABC}}}\mathcal{D}^{ \mathfrak{\mathrm{\alpha }}}_{\mathrm{t}}\mathrm{u}(\mathrm{t})) + \Psi (\mathrm{t})$.

### Definition 6

(Hyers–Ulam–Rassias stable)

Our BVP is Hyers–Ulam–Rassias stable with respect to $\mathfrak{\psi } \in \mathbb{ X} $ if there exists a real number $\mathcal{C}_{\mathrm{g}} > 0 $ such that, for $\epsilon > 0 $ and for any solution $\mathrm{u}\in \mathbb{X} $ of the inequality

18 $$\begin{aligned} \bigl\vert {}_{0}^{{\mathcal{ABC}}}\mathcal{D}^{\mathfrak{\mathrm{\alpha }}}_{ \mathrm{t}} \mathrm{u}(\mathrm{t}) - \mathrm{g}\bigl(\mathrm{t}, \mathrm{u} (\mathrm{t}), _{0}^{{\mathcal{ABC}}}\mathcal{D}^{ \mathfrak{\mathrm{\alpha }}}_{\mathrm{t}}\mathrm{u}( \mathrm{t})\bigr) \bigr\vert \leq \mathfrak{\psi }(\mathrm{t})\epsilon, \end{aligned}$$

there exists the unique solution $\mathrm{u}^{\star }\in \mathbb{X}$ for our BVP (3) such that

$$\begin{aligned} \bigl\Vert \mathrm{u}-\mathrm{u}^{\star } \bigr\Vert \leq \mathcal{C}_{\mathrm{g}} \mathfrak{\psi }(\mathrm{t}) (\epsilon ). \end{aligned}$$

### Definition 7

(Generalized Hyers–Ulam–Rassias stable)

Our BVP (3) is generalized Hyers–Ulam–Rassias stable with respect to $\mathfrak{\psi } \in \mathcal{C}[0, 1] $, if there is $\mathcal{C}_{\mathrm{g}} > 0 $ such that, for any solution $\mathrm{u} \in \mathbb{X}$ of inequality (18), there exists the unique solution $\mathrm{u}^{\star }\in \mathbb{X}$ of our BVP such that

$$\begin{aligned} \bigl\Vert \mathrm{u}-\mathrm{u}^{\star } \bigr\Vert \leq \mathcal{C}_{\mathrm{g}} \mathfrak{\psi }(\mathrm{t}). \end{aligned}$$

### Remark 2

A function $\mathrm{u}\in \mathbb{X} $ is the solution of our inequality (18) if there is a function $\Psi \in \mathcal{C}[0, 1] $ that depends on u such that

$(1)$ $|\Psi (\mathrm{t})| \leq \mathfrak{\psi }(\mathrm{t}) \epsilon $

$(2)$ ${}_{0}^{{\mathcal{ABC}}}\mathcal{D}^{\mathfrak{\mathrm{\alpha }}}_{ \mathrm{t}} \mathrm{u}(\mathrm{t}) = \mathrm{g}(\mathrm{t}, \mathrm{u} (\mathrm{t}),{}_{0}^{{\mathcal{ABC}}}\mathcal{D}^{ \mathfrak{\mathrm{\alpha }}}_{\mathrm{t}}\mathrm{u}(\mathrm{t})) + \Psi (\mathrm{t})$.

### Lemma 3

*By using Remark *1, *the function* $\mathrm{u}\in \mathbb{X} $ *corresponding to the given problem*

19 $$\begin{aligned} \textstyle\begin{cases} {{}_{0}^{{\mathcal{ABC}}}\mathcal{D}^{\mathfrak{\mathrm{\alpha }}}_{\xi } \mathrm{u}(\mathrm{t})} = \mathrm{g}(\mathrm{t}, \mathrm{u} ( \mathrm{t}),{}_{0}^{{\mathcal{ABC}}}\mathcal{D}^{ \mathfrak{\mathrm{\alpha }}}_{\xi }\mathrm{u}(\mathrm{t})) + \Psi ( \mathrm{t}),\quad 1< {\mathfrak{\mathrm{\alpha }}}\leq 2, \mathrm{t}\in \mathcal{J}, \\ \mathrm{u}(0)=\mathrm{u}_{0},\qquad \mathrm{u}(\mathfrak{\mathrm{b}})= \mathrm{u}_{1}. \end{cases}\displaystyle \end{aligned}$$

*satisfies the inequality*

20 $$\begin{aligned} \bigl\vert \mathrm{u}(\mathrm{t}) - \mathcal{H}\mathrm{u}( \mathrm{t}) \bigr\vert \leq \mathcal{C}_{\mathrm{g} \mathfrak{\mathrm{\alpha }}}\epsilon,\quad \forall \mathrm{t} \in \mathcal{J}. \end{aligned}$$

*Now*, *one has*

$$\begin{aligned} \begin{aligned} \mathcal{H}\mathrm{u}(\mathrm{t})={}& \frac{\mathrm{t}\mathrm{u}_{1} + \mathrm{u}_{0}(\mathfrak{\mathrm{b}}-\mathrm{t})}{\mathfrak{\mathrm{b}}} - \frac{\mathrm{t}(2-{\mathfrak{\mathrm{\alpha }}})}{\mathfrak{\mathrm{b}} \mathbb{M}({\mathfrak{\mathrm{\alpha }}}-1)} \int _{0}^{\mathfrak{\mathrm{b}}} \mathrm{g}\bigl(\xi, \mathrm{u}(\xi ), {}_{0}^{{\mathcal{ABC}}}\mathcal{D}^{\mathfrak{\mathrm{\alpha }}}_{\xi } \mathrm{u}(\xi )\bigr) \,d\xi \\ &{} - \frac{\mathrm{t}({\mathfrak{\mathrm{\alpha }}}-1)}{\mathfrak{\mathrm{b}}\mathbb{M}({\mathfrak{\mathrm{\alpha }}}-1)\Gamma ({\mathfrak{\mathrm{\alpha }}})} \int _{0}^{\mathfrak{\mathrm{b}}}\mathrm{g}\bigl(\xi, \mathrm{u}(\xi ),{}_{0}^{{ \mathcal{ABC}}}\mathcal{D}^{\mathfrak{\mathrm{\alpha }}}_{\xi } \mathrm{u}(\xi )\bigr) (\mathfrak{\mathrm{b}}-\xi )^{{ \mathfrak{\mathrm{\alpha }}}-1}\,d\xi \\ &{} + \frac{2-{\mathfrak{\mathrm{\alpha }}}}{\mathbb{M}({\mathfrak{\mathrm{\alpha }}}-1)} \int _{0}^{\mathrm{t}} \mathrm{g}\bigl(\xi, \mathrm{u}(\xi ),{}_{0}^{{ \mathcal{ABC}}}\mathcal{D}^{\mathfrak{\mathrm{\alpha }}}_{\xi } \mathrm{u}(\xi )\bigr) \,d\xi \\ &{} + \frac{{\mathfrak{\mathrm{\alpha }}}-1}{\mathbb{M}({\mathfrak{\mathrm{\alpha }}}-1)\Gamma ({\mathfrak{\mathrm{\alpha }}})} \int _{0}^{\mathrm{t}} \mathrm{g}\bigl(\xi, \mathrm{u}(\xi ),{}_{0}^{{ \mathcal{ABC}}}\mathcal{D}^{\mathfrak{\mathrm{\alpha }}}_{\xi } \mathrm{u}(\xi )\bigr) (\mathrm{t}-\xi )^{{\mathfrak{\mathrm{\alpha }}}-1}\,d \xi, \end{aligned} \end{aligned}$$

*and we have*

$$\begin{aligned} \mathcal{C}_{\mathrm{g}, \mathfrak{\mathrm{\alpha }}} = \frac{4(\mathfrak{\mathrm{b}}\Gamma ({\mathfrak{\mathrm{\alpha }}}+1)+ \mathfrak{\mathrm{b}}^{{\mathfrak{\mathrm{\alpha }}}})}{\mathbb{M}({\mathfrak{\mathrm{\alpha }}}-1)\Gamma ({\mathfrak{\mathrm{\alpha }}}+1)} . \end{aligned}$$

### Proof

In view of Lemma 2 and equation (19), we get

21 $$\begin{aligned} &\begin{aligned} \mathrm{u}(\mathrm{t}) ={}& \mathfrak{c}_{0}+\mathfrak{c}_{1}\mathrm{t}+ \frac{2-{\mathfrak{\mathrm{\alpha }}}}{\mathbb{M}({\mathfrak{\mathrm{\alpha }}}-1)} \int _{0}^{\mathrm{t}} \bigl(\mathrm{g}\bigl(\xi, \mathrm{u} (\xi ),{}_{0}^{{ \mathcal{ABC}}}\mathcal{D}^{\mathfrak{\mathrm{\alpha }}}_{\xi } \mathrm{u}(\xi )\bigr) + \Psi (\mathrm{t})\bigr) \,d\xi \\ &{}+ \frac{{\mathfrak{\mathrm{\alpha }}}-1}{\mathbb{M}({\mathfrak{\mathrm{\alpha }}}-1) \Gamma ({\mathfrak{\mathrm{\alpha }}})} \int _{0}^{\mathrm{t}} \bigl( \mathrm{g}\bigl(\xi, \mathrm{u} (\xi ),{}_{0}^{{\mathcal{ABC}}} \mathcal{D}^{\mathfrak{\mathrm{\alpha }}}_{\xi } \mathrm{u}(\xi )\bigr) + \Psi (\mathrm{t})\bigr) (\mathrm{t}-\xi )^{{\mathfrak{\mathrm{\alpha }}}-1} \,d \xi, \end{aligned} \\ &\begin{aligned} \mathrm{u}(\mathrm{t}) ={}& \frac{\mathrm{t}\mathrm{u}_{1} + \mathrm{u}_{0}(\mathfrak{\mathrm{b}}-\mathrm{t})}{\mathfrak{\mathrm{b}}}- \frac{\mathrm{t}(2-{\mathfrak{\mathrm{\alpha }}})}{\mathfrak{\mathrm{b}} \mathbb{M}({\mathfrak{\mathrm{\alpha }}}-1)} \int _{0}^{\mathfrak{\mathrm{b}}} \mathrm{g}\bigl(\xi, \mathrm{u}(\xi ), {}_{0}^{{\mathcal{ABC}}}\mathcal{D}^{\mathfrak{\mathrm{\alpha }}}_{\xi } \mathrm{u} (\xi )\bigr) \,d\xi \\ &{}- \frac{\mathrm{t}({\mathfrak{\mathrm{\alpha }}}-1)}{\mathfrak{\mathrm{b}}\mathbb{M}({\mathfrak{\mathrm{\alpha }}}-1)\Gamma ({\mathfrak{\mathrm{\alpha }}})} \int _{0}^{\mathfrak{\mathrm{b}}}\mathrm{g}\bigl(\xi, \mathrm{u}(\xi ), _{0}^{{ \mathcal{ABC}}}\mathcal{D}^{\mathfrak{\mathrm{\alpha }}}_{\xi } \mathrm{u}(\xi )\bigr) (\mathfrak{\mathrm{b}}-\xi )^{ \mathfrak{\mathrm{\alpha }-1}}\,d\xi \\ &{}+ \frac{2-{\mathfrak{\mathrm{\alpha }}}}{\mathbb{M}({\mathfrak{\mathrm{\alpha }}}-1)} \int _{0}^{\mathrm{t}} \mathrm{g}\bigl(\xi, \mathrm{u} (\xi ),{}_{0}^{{ \mathcal{ABC}}}\mathcal{D}^{\mathfrak{\mathrm{\alpha }}}_{\xi } \mathrm{u}(\xi )\bigr) \,d\xi \\ &{}+ \frac{{\mathfrak{\mathrm{\alpha }}}-1}{\mathbb{M}({\mathfrak{\mathrm{\alpha }}}-1)\Gamma ({\mathfrak{\mathrm{\alpha }}})} \int _{0}^{\mathrm{t}} \mathrm{g}\bigl(\xi, \mathrm{u} (\xi ),{}_{0}^{{ \mathcal{ABC}}}\mathcal{D}^{\mathfrak{\mathrm{\alpha }}}_{\xi } \mathrm{u}(\xi )\bigr) (\mathrm{t}-\xi )^{{\mathfrak{\mathrm{\alpha }}}-1} \,d \xi \\ &{} - \frac{\mathrm{t}(2-{\mathfrak{\mathrm{\alpha }}})}{\mathfrak{\mathrm{b}} \mathbb{M}({\mathfrak{\mathrm{\alpha }}}-1)} \int _{0}^{\mathfrak{\mathrm{b}}}\Psi (\xi ) \,d\xi - \frac{\mathrm{t}({\mathfrak{\mathrm{\alpha }}}-1)}{\mathfrak{\mathrm{b}}\mathbb{M}({\mathfrak{\mathrm{\alpha }}}-1)\Gamma ({\mathfrak{\mathrm{\alpha }}})} \int _{0}^{\mathfrak{\mathrm{b}}}\Psi (\xi ) (\mathfrak{\mathrm{b}}- \xi )^{\mathfrak{\mathrm{\alpha }-1}}\,d\xi \\ &{}+ \frac{2-{\mathfrak{\mathrm{\alpha }}}}{\mathbb{M}({\mathfrak{\mathrm{\alpha }}}-1)} \int _{0}^{\mathrm{t}} \Psi (\xi ) \,d\xi + \frac{{\mathfrak{\mathrm{\alpha }}}-1}{\mathbb{M}({\mathfrak{\mathrm{\alpha }}}-1)\Gamma ({\mathfrak{\mathrm{\alpha }}})} \int _{0}^{\mathrm{t}} \Psi (\xi ) (\mathrm{t}-\xi )^{{ \mathfrak{\mathrm{\alpha }}}-1} \,d\xi. \end{aligned} \end{aligned}$$

Now from the above equation we conclude our result as follows:

$$\begin{aligned} \bigl\vert \mathrm{u}(\mathrm{t}) - \mathcal{H}\mathrm{u}(\mathrm{t}) \bigr\vert \leq \mathcal{C}_{\mathrm{g}, \mathfrak{\mathrm{\alpha }}}\epsilon. \end{aligned}$$

 □

### Theorem 4

*In view of assumption* $(\mathrm{A}_{1}) $ *and Lemma *3, *the solution of our boundary value problem is Hyers–Ulam stable and generalized Hyers–Ulam stable if* $4 \mathrm{g}_{\mathrm{g}}\mathcal{C}_{\mathrm{g}, \mathfrak{\mathrm{\alpha }}} < 1$.

### Proof

If $\mathrm{u}^{\star }(\mathrm{t})$ is the unique solution and $\mathrm{u}(\mathrm{t})$ is any solution of our modeled problem, then we have

$$\begin{aligned} \bigl\vert \mathrm{u}(\mathrm{t})- \mathrm{u}^{\star }(\mathrm{t}) \bigr\vert &= \bigl\vert \mathrm{u}( \mathrm{t}) - \mathcal{H}\mathrm{u}^{\star }(\mathrm{t}) \bigr\vert \\ & = \bigl\vert \mathrm{u}(\mathrm{t}) - \mathcal{H}\mathrm{u}(\mathrm{t}) + \mathcal{H}\mathrm{u}(\mathrm{t})-\mathcal{H}\mathrm{u}^{\star }( \mathrm{t}) \bigr\vert \\ & \leq \bigl\vert \mathrm{u}(\mathrm{t}) - \mathcal{H}\mathrm{u}(\mathrm{t}) \bigr\vert + \bigl\vert \mathcal{H}\mathrm{u}(\mathrm{t}) - \mathcal{H} \mathrm{u}^{\star }(\mathrm{t}) \bigr\vert \\ &\leq \mathcal{C}_{\mathrm{g}, \mathfrak{\mathrm{\alpha }}}\epsilon + \bigl\vert \mathcal{H}\mathrm{u}( \mathrm{t})-\mathcal{H}\mathrm{u}( \mathrm{t}) \bigr\vert \\ & \leq \mathcal{C}_{\mathrm{g}, \mathfrak{\mathrm{\alpha }}}\epsilon + 4 \biggl(\mathcal{L}_{\mathrm{g}} + \frac{\mathcal{L}_{\mathrm{g}} ^{2}}{1-\mathcal{L}_{\mathrm{g}} } \biggr) \frac{\mathfrak{\mathrm{b}}\Gamma ({\mathfrak{\mathrm{\alpha }}}+1)+ \mathfrak{\mathrm{b}}^{\mathfrak{\mathrm{\alpha }}}}{\mathbb{M}({\mathfrak{\mathrm{\alpha }}}-1)\Gamma ({\mathfrak{\mathrm{\alpha }}}+1)} \bigl\Vert \mathrm{u} - \mathrm{u}^{\star } \bigr\Vert \\ & \leq \mathcal{C}_{\mathrm{g}, \mathfrak{\mathrm{\alpha }}}\epsilon + 4 \mathrm{g}_{\mathrm{g}} \mathcal{C}_{\mathrm{g}, \mathfrak{\mathrm{\alpha }}} \bigl\Vert \mathrm{u}- \mathrm{u}^{\star } \bigr\Vert , \end{aligned}$$

where

$$\begin{aligned} \mathrm{g}_{\mathrm{g}} = \biggl(\mathcal{L}_{\mathrm{g}} + \frac{\mathcal{L}_{\mathrm{g}} ^{2}}{1-\mathcal{L}_{\mathrm{g}} } \biggr). \end{aligned}$$

From above we have

$$\begin{aligned} \bigl\Vert \mathrm{u}- \mathrm{u}^{\star } \bigr\Vert \leq \frac{\mathcal{C}_{\mathrm{g}, \mathfrak{\mathrm{\alpha }}}\epsilon }{1 - 4 \mathrm{g}_{\mathrm{g}}\mathcal{C}_{\mathrm{g}, \mathfrak{\mathrm{\alpha }}}}. \end{aligned}$$

Let

$$\begin{aligned} &\mathfrak{E}_{\mathrm{g}}= \frac{\mathcal{C}_{\mathrm{g}, \mathfrak{\mathrm{\alpha }}} }{1 - 4 \mathrm{g}_{\mathrm{g}}\mathcal{C}_{\mathrm{g}, \mathfrak{\mathrm{\alpha }}}} \\ &\bigl\Vert \mathrm{u}- \mathrm{u}^{\star } \bigr\Vert \leq \mathfrak{E}_{\mathrm{g}} \epsilon. \end{aligned}$$

Thus our solution is Hyers–Ulam stable. And if $\mathfrak{\psi }(\epsilon )=\epsilon $, thus our problem is also generalized Hyers–Ulam stable. □

### Lemma 4

*In view of Remark *2, *the BVP mentioned in Lemma *3*satisfies the inequality*

$$\begin{aligned} \bigl\vert \mathrm{u}(\mathrm{t}) - \mathcal{H}\bigl(\mathrm{t}, \mathrm{u} ( \mathrm{t}),{}_{0}^{{\mathcal{ABC}}}\mathcal{D}^{ \mathfrak{\mathrm{\alpha }}}_{\mathfrak{\mathrm{t}}} \mathrm{u}( \mathrm{t})\bigr) \bigr\vert \leq \mathcal{C}_{\mathrm{g}, \mathfrak{\mathrm{\alpha }}}\epsilon \Psi (\mathrm{t})\quad \forall \mathrm{t} \in [0, \mathfrak{\mathrm{b}}]. \end{aligned}$$

### Proof

We prove this inequality in view of Remark 2, calculations are the same as in Lemma 3. □

### Theorem 5

*In view of Lemma *4*and under consideration of assumption* $\mathrm{A}_{1} $, *the solution of our problem is Hyers–Ulam–Rassias stable and generalized Hyers–Ulam–Rassias stable if* $4 \mathrm{g}_{\mathrm{g}}\mathcal{C}_{\mathrm{g}, \mathfrak{\mathrm{\alpha }}} < 1$.

### Proof

22 $$\begin{aligned} \bigl\vert \mathrm{u}(\mathrm{t})- \mathrm{u}^{\star }(\mathrm{t}) \bigr\vert & = \bigl\vert \mathrm{u}( \mathrm{t}) - \mathcal{H}\mathrm{u}^{\star }(\mathrm{t}) \bigr\vert \\ & =\bigl\vert \mathrm{u}(\mathrm{t}) - \mathcal{H}\mathrm{u}(\mathrm{t}) + \mathcal{H}\mathrm{u}(\mathrm{t})-\mathcal{H}\mathrm{u}^{\star }( \mathrm{t}) \bigr\vert \\ & \leq \bigl\vert \mathrm{u}(\mathrm{t}) - \mathcal{H}\mathrm{u}( \mathrm{t}) \bigr\vert + \bigl\vert \mathcal{H}\mathrm{u}(\mathrm{t})- \mathcal{H} \mathrm{u}^{\star }(\mathrm{t}) \bigr\vert \\ & \leq \mathcal{C}_{\mathrm{g}, \mathfrak{\mathrm{\alpha }}}\epsilon \mathfrak{\psi }(\mathrm{t}) + \bigl\vert \mathcal{H}\mathrm{u}(\mathrm{t})- \mathcal{H}\mathrm{u}^{\star }( \mathrm{t}) \bigr\vert \\ &\leq \mathcal{C}_{\mathrm{g}, \mathfrak{\mathrm{\alpha }}}\epsilon \mathfrak{\psi }(\mathrm{t}) + 4 \mathrm{g}_{\mathrm{g}}\mathcal{C}_{ \mathrm{g}, \mathfrak{\mathrm{\alpha }}} \bigl\Vert \mathrm{u}- \mathrm{u}^{ \star } \bigr\Vert . \end{aligned}$$

After simplification, from (22), one has

$$\begin{aligned} \bigl\Vert \mathrm{u}- \mathrm{u}^{\star } \bigr\Vert \leq \frac{\mathcal{C}_{\mathrm{g}, \mathfrak{\mathrm{\alpha }}}}{1-4 \mathrm{g}_{\mathrm{g}}\mathcal{C}_{\mathrm{g}, \mathfrak{\mathrm{\alpha }}}} \epsilon \mathfrak{\psi }(\mathrm{t}). \end{aligned}$$

Thus our problem is generalized Hyers–Ulam–Rassias stable. □

## Illustrative examples

In this section, we verify our boundary value problem with different examples.

### Example 1

Considered the following BVP:

23 $$\begin{aligned} \textstyle\begin{cases} {{}_{0}^{{\mathcal{ABC}}}\mathcal{D}^{\frac{3}{2}}_{\mathrm{t}} \mathrm{u}(\mathrm{t})} = \frac{\mathrm{t} \ln {\mathrm{t}}}{20} + \frac{\mathrm{u}(\mathrm{t})e^{-\sin {\mathrm{t}}}}{90} + \frac{{_{0}^{{\mathcal{ABC}}}\mathcal{D}^{\mathfrak{\mathrm{\alpha }}}_{\mathrm{t}}\mathrm{u}(\frac{\mathrm{t}}{3})}}{90 e^{-\mathrm{t}-\cos \mathrm{t}}}, \quad \mathrm{t}\in [0, 1], \\ \mathrm{u}(0)=0, \qquad \mathrm{u}(1)=0. \end{cases}\displaystyle \end{aligned}$$

Here, $\mathfrak{\mathrm{b}}= 1 $ and ${\mathfrak{\mathrm{\alpha }}}= \frac{3}{2} $

$$\begin{aligned} \mathrm{g}(\mathrm{t}, \mathrm{u}, \mathrm{v})= \frac{\mathrm{t} \ln {\mathrm{t}}}{20} + \frac{\mathrm{u}(\mathrm{t})e^{-\sin {\mathrm{t}}}}{90} + \frac{{_{0}^{{\mathcal{ABC}}}\mathcal{D}^{\mathfrak{\mathrm{\alpha }}}_{\mathrm{t}}\mathrm{u}(\frac{\mathrm{t}}{3})}}{90 e^{-\mathrm{t}-\cos \mathrm{t}}}. \end{aligned}$$

Let $\mathrm{u}, \overline{\mathrm{u}}, \mathrm{v}, \overline{\mathrm{v}} \in \mathbb{X} $

$$\begin{aligned} &\bigl\vert \mathrm{g} (\mathrm{t}, \mathrm{u}, \mathrm{v})-\mathrm{g}( \mathrm{t}, \overline{\mathrm{u}}, \overline{\mathrm{v}}) \bigr\vert \\ &\quad = \biggl\vert \biggl[\frac{\mathrm{t} \ln {\mathrm{t}}}{20} + \frac{\mathrm{u}(\mathrm{t})e^{-\sin {\mathrm{t}}}}{90} + \frac{{_{0}^{{\mathcal{ABC}}}\mathcal{D}^{\frac{3}{2}}_{\mathrm{t}}\mathrm{u}(\frac{\mathrm{t}}{3})}}{90 e^{-\mathrm{t}-\cos \mathrm{t}}} \biggr] - \biggl[\frac{\mathrm{t} \ln {\mathrm{t}}}{20} + \frac{\overline{\mathrm{u}}(\mathrm{t})e^{-\sin {\mathrm{t}}}}{90} + \frac{{_{0}^{{\mathcal{ABC}}}\mathcal{D}^{\frac{3}{2}}_{\mathrm{t}}{\overline{\mathrm{u}}(\frac{\mathrm{t}}{3})}}}{90 e^{-\mathrm{t}-\cos \mathrm{t}}} \biggr] \biggr\vert \\ &\quad\leq \frac{1}{90} \vert \mathrm{u}-\overline{\mathrm{u}} \vert + \frac{1}{90} \vert \mathrm{v}-\overline{\mathrm{v}} \vert . \end{aligned}$$

Now, using assumption $(\mathrm{A}_{2}) $, we obtain

$$\begin{aligned} \bigl\vert \mathrm{g}(\mathrm{t}, \mathrm{u}, \mathrm{v}) \bigr\vert &= \biggl\vert \frac{\mathrm{t} \ln {\mathrm{t}}}{20} + \frac{\mathrm{u}(\mathrm{t})e^{-\sin {\mathrm{t}}}}{90} + \frac{{_{0}^{{\mathcal{ABC}}}\mathcal{D}^{\frac{3}{2}}_{\mathrm{t}}\mathrm{u}(\frac{\mathrm{t}}{3})}}{90 e^{-\mathrm{t}-\cos \mathrm{t}}} \biggr\vert \\ &\leq \frac{1}{20}+\frac{1}{90} \vert \mathrm{u} \vert + \frac{1}{90} \vert \mathrm{v} \vert . \end{aligned}$$

Thus, we have $\mathcal{L}_{\mathrm{g}}=\frac{1}{90} $, ${\mathfrak{\mathrm{\alpha }}} = \frac{3}{2}, \mathbf{A}_{\mathrm{g}}= \frac{1}{20}, \mathbf{B}_{\mathrm{g}} = \frac{1}{90}$, and $\mathbf{C}_{\mathrm{g}} = \frac{1}{90} $

$$\begin{aligned} 4 \biggl(\mathcal{L}_{\mathrm{g}} + \frac{\mathcal{L}_{\mathrm{g}} ^{2}}{1-\mathcal{L}_{\mathrm{g}} } \biggr) \frac{\mathfrak{\mathrm{b}}\Gamma ({\mathfrak{\mathrm{\alpha }}}+1)+ \mathfrak{\mathrm{b}}^{\mathfrak{\mathrm{\alpha }}}}{\mathbb{M}({\mathfrak{\mathrm{\alpha }}}-1)\Gamma ({\mathfrak{\mathrm{\alpha }}}+1)}= 0.008440< 1. \end{aligned}$$

Thus all the conditions for Theorem 2 are satisfied. Hence, in view of equation (9), our BVP has the unique solution. Also

$$\begin{aligned} 2 \biggl(\mathcal{L}_{\mathrm{g}} + \frac{\mathcal{L}_{\mathrm{g}} ^{2}}{1-\mathcal{L}_{\mathrm{g}} } \biggr) \frac{\mathfrak{\mathrm{b}}\Gamma ({\mathfrak{\mathrm{\alpha }}}+1) + \mathfrak{\mathrm{b}}^{\mathfrak{\mathrm{\alpha }}}}{\mathbb{M}({\mathfrak{\mathrm{\alpha }}}-1)\Gamma ({\mathfrak{\mathrm{\alpha }}}+1)}= 0.0168959 < 1. \end{aligned}$$

The condition for Theorem 3 is also satisfied. Moreover, we also have $\mathcal{C}_{\mathrm{g}, \mathfrak{\mathrm{\alpha }}}\neq 1$. Hence the solution is both Hyers–Ulam and generalized Hyers–Ulam stable. In a similar way, one can easily verify the conditions of Hyers–Ulam–Rassias and generalized Hyers–Ulam–Rassias stability.

### Example 2

Consider the following nonhomogeneous BVP:

24 $$\begin{aligned} \textstyle\begin{cases} {{}_{0}^{{\mathcal{ABC}}}\mathcal{D}^{\frac{4}{3}}_{\mathrm{t}} \mathrm{u}(\mathrm{t})} = \frac{\mathrm{t}^{2}+ e^{\ln {\mathrm{t}}+\mathrm{t}}}{25} + \frac{e^{\sin \mathrm{t} \cos \mathrm{t}}\mathrm{u}(\mathrm{t})}{60} + \frac{e^{\mathrm{t}^{2}-\cos ^{2} \mathrm{t}}{_{0}^{{\mathcal{ABC}}}\mathcal{D}^{\frac{4}{3}}_{\mathrm{t}}}\mathrm{u}(\mathrm{t})}{60}, \quad \mathrm{t}\in [0, 1], \\ \mathrm{u}(0)= e^{2\pi }, \qquad \mathrm{u}(1)=\sin \mathrm{u}( \frac{2\pi }{3}). \end{cases}\displaystyle \end{aligned}$$

Here, $\mathfrak{\mathrm{b}} = 1 $ and ${\mathfrak{\mathrm{\alpha }}} = \frac{4}{3} $

$$\begin{aligned} \mathrm{g}(\mathrm{t}, \mathrm{u}, \mathrm{v})= \frac{\mathrm{t}^{2}+ e^{\ln {\mathrm{t}}+\mathrm{t}}}{25} + \frac{e^{\sin \mathrm{t} \cos \mathrm{t}}\mathrm{u}(\mathrm{t})}{60} + \frac{e^{\mathrm{t}^{2}-\cos ^{2} \mathrm{t}}{_{0}^{{\mathcal{ABC}}}\mathcal{D}^{\frac{4}{3}}_{\mathrm{t}}}\mathrm{u}(\mathrm{t})}{60}. \end{aligned}$$

Let $\mathrm{u}, \overline{\mathrm{u}}, \mathrm{v}, \overline{\mathrm{v}} \in \mathbb{X} $

$$\begin{aligned} \begin{aligned} ={}& \bigl\vert \mathrm{g} (\mathrm{t}, \mathrm{u}, \mathrm{v})-\mathrm{g}( \mathrm{t}, \overline{\mathrm{u}}, \overline{\mathrm{v}}) \bigr\vert \\ ={}& \biggl\vert \biggl[ \frac{\mathrm{t}^{2}+ e^{\ln {\mathrm{t}}+\mathrm{t}}}{25} + \frac{e^{\sin \mathrm{t} \cos \mathrm{t}}\mathrm{u}(\mathrm{t})}{60} + \frac{e^{\mathrm{t}^{2}-\cos ^{2}}{_{0}^{{\mathcal{ABC}}}\mathcal{D}^{\frac{4}{3}}_{\mathrm{t}}}\mathrm{u}(\mathrm{t})}{60} \biggr] \\ &{} - \biggl[ \frac{\mathrm{t}^{2}+ e^{\ln {\mathrm{t}}+\mathrm{t}}}{25} + \frac{e^{\sin \mathrm{t} \cos \mathrm{t}}\mathrm{u}(\mathrm{t})}{60} + \frac{e^{\mathrm{t}^{2}-\cos ^{2} \mathrm{t}}{_{0}^{{\mathcal{ABC}}}\mathcal{D}^{\frac{4}{3}}_{\mathrm{t}}}\mathrm{u}(\mathrm{t})}{60} \biggr] \biggr\vert \\ \leq{}& \frac{1}{60} \vert \mathrm{u}-\overline{\mathrm{u}} \vert + \frac{1}{60} \vert \mathrm{v}-\overline{\mathrm{v}} \vert . \end{aligned} \end{aligned}$$

Now, one has

$$\begin{aligned} \bigl\vert \mathrm{g}(\mathrm{t}, \mathrm{u}, \mathrm{v}) \bigr\vert &= \biggl\vert \frac{\mathrm{t}^{2}+ e^{\ln {\mathrm{t}}+\mathrm{t}}}{25} + \frac{e^{\sin \mathrm{t} \cos \mathrm{t}}\mathrm{u}(\mathrm{t})}{60} + \frac{e^{\mathrm{t}^{2}-\cos ^{2} \mathrm{t}}{_{0}^{{\mathcal{ABC}}}\mathcal{D}^{\frac{4}{3}}_{\mathrm{t}}}\mathrm{u}(\mathrm{t})}{60} \biggr\vert . \\ &\leq \frac{1}{25}+\frac{1}{60} \vert \mathrm{u} \vert + \frac{1}{60} \vert \mathrm{v} \vert . \end{aligned}$$

We have

$$\begin{aligned} \mathcal{L}_{\mathrm{g}}=\frac{1}{60},\qquad {\mathfrak{\mathrm{\alpha }}} = \frac{4}{3}, \qquad\mathbf{A}_{\mathrm{g}} = \frac{1}{25},\qquad \mathbf{B}_{ \mathrm{g}} = \frac{1}{60}\quad \text{and} \quad\mathbf{C}_{\mathrm{g}}= \frac{1}{60}. \end{aligned}$$

Thus

$$\begin{aligned} 4 \biggl(\mathcal{L}_{\mathrm{g}} + \frac{\mathcal{L}_{\mathrm{g}} ^{2}}{1-\mathcal{L}_{\mathrm{g}} } \biggr) \frac{\mathfrak{\mathrm{b}}\Gamma ({\mathfrak{\mathrm{\alpha }}}+1)+ \mathfrak{\mathrm{b}}^{\mathfrak{\mathrm{\alpha }}}}{\mathbb{M}({\mathfrak{\mathrm{\alpha }}}-1)\Gamma ({\mathfrak{\mathrm{\alpha }}}+1)} < 1. \end{aligned}$$

Therefore, all the conditions for Theorem 2 are satisfied. Hence, in view of equation (9), the given problem has the unique solution. Also

$$\begin{aligned} 2 \biggl(\mathcal{L}_{\mathrm{g}} + \frac{\mathcal{L}_{\mathrm{g}} ^{2}}{1-\mathcal{L}_{\mathrm{g}} } \biggr) \frac{\mathfrak{\mathrm{b}}\Gamma ({\mathfrak{\mathrm{\alpha }}}+1)+ \mathfrak{\mathrm{b}}^{\mathfrak{\mathrm{\alpha }}}}{\mathbb{M}({\mathfrak{\mathrm{\alpha }}}-1)\Gamma ({\mathfrak{\mathrm{\alpha }}}+1)} < 1. \end{aligned}$$

The condition for Theorem 3 is also satisfied. Moreover, we also have $\mathcal{C}_{\mathrm{g}, \mathfrak{\mathrm{\alpha }}}\neq 1$. Hence the solution is both Hyers–Ulam and generalized Hyers–Ulam stable. In a similar way one can easily verify the conditions of Hyers–Ulam–Rassias and generalized Hyers–Ulam–Rassias stability.

### Example 3

Here we take another BVP as follows:

25 $$\begin{aligned} \textstyle\begin{cases} {{}_{0}^{{\mathcal{ABC}}}\mathcal{D}^{\frac{3}{2}}_{\mathrm{t}} \mathrm{u}(\mathrm{t})} = \frac{\sin (\mathrm{t})}{\mathrm{t}+50} + \frac{e^{-\mathrm{t}}\mathrm{u}(\mathrm{t})}{100} + \frac{e^{-\mathrm{t}^{2}}{_{0}^{{\mathcal{ABC}}}\mathcal{D}^{\frac{3}{2}}_{\mathrm{t}}\mathrm{u}(\mathrm{t})}}{100},\quad \mathrm{t}\in [0, 1], \\ \mathrm{u}(0)= 1, \qquad \mathrm{u}(1)=2. \end{cases}\displaystyle \end{aligned}$$

One has $\mathfrak{\mathrm{b}} = 1 $ and ${\mathfrak{\mathrm{\alpha }}} = \frac{3}{2} $

$$\begin{aligned} \mathrm{g}(\mathrm{t}, \mathrm{u}, \mathrm{v})= \frac{\sin (\mathrm{t})}{\mathrm{t}+50} + \frac{e^{-\mathrm{t}}\mathrm{u}(\mathrm{t})}{100} + \frac{e^{-\mathrm{t}^{2}}{_{0}^{{\mathcal{ABC}}}\mathcal{D}^{\frac{3}{2}}_{\mathrm{t}}\mathrm{u}(\mathrm{t})}}{100}. \end{aligned}$$

Let $\mathrm{u}, \overline{\mathrm{u}}, \mathrm{v}, \overline{\mathrm{v}} \in \mathbb{X}$, one has

$$\begin{aligned} \bigl\vert \mathrm{g} (\mathrm{t}, \mathrm{u}, \mathrm{v})-\mathrm{g}( \mathrm{t}, \overline{\mathrm{u}}, \overline{\mathrm{v}}) \bigr\vert \leq \frac{1}{100} \vert \mathrm{u}-\overline{\mathrm{u}} \vert + \frac{1}{100} \vert \mathrm{v}-\overline{\mathrm{v}} \vert . \end{aligned}$$

Now

$$\begin{aligned} \bigl\vert \mathrm{g}(\mathrm{t}, \mathrm{u}, \mathrm{v}) \bigr\vert &= \biggl\vert \frac{\sin (\mathrm{t})}{\mathrm{t}+50} + \frac{e^{-\mathrm{t}}\mathrm{u}(\mathrm{t})}{100} + \frac{e^{-\mathrm{t}^{2}}{_{0}^{{\mathcal{ABC}}}\mathcal{D}^{\frac{3}{2}}_{\mathrm{t}}\mathrm{u}(\mathrm{t})}}{100} \biggr\vert \\ &\leq \frac{1}{50}+\frac{1}{100} \vert \mathrm{u} \vert + \frac{1}{100} \vert \mathrm{v} \vert . \end{aligned}$$

We have

$$\begin{aligned} \mathcal{L}_{\mathrm{g}}=\frac{1}{100},\qquad{\mathfrak{\mathrm{\alpha }}} = \frac{3}{2}, \qquad\mathbf{A}_{\mathrm{g}} = \frac{1}{50},\qquad \mathbf{B}_{ \mathrm{g}} = \frac{1}{100} \quad\text{and}\quad \mathbf{C}_{\mathrm{g}}= \frac{1}{100}. \end{aligned}$$

Therefore, we have

$$\begin{aligned} 4 \biggl(\mathcal{L}_{\mathrm{g}} + \frac{\mathcal{L}_{\mathrm{g}} ^{2}}{1-\mathcal{L}_{\mathrm{g}} } \biggr) \frac{\mathfrak{\mathrm{b}}\Gamma ({\mathfrak{\mathrm{\alpha }}}+1)+ \mathfrak{\mathrm{b}}^{\mathfrak{\mathrm{\alpha }}}}{\mathbb{M}({\mathfrak{\mathrm{\alpha }}}-1)\Gamma ({\mathfrak{\mathrm{\alpha }}}+1)} < 1. \end{aligned}$$

Hence all the conditions of Theorem 2 are satisfied. Further, the condition for uniqueness is also satisfied. Also

$$\begin{aligned} 2 \biggl(\mathcal{L}_{\mathrm{g}} + \frac{\mathcal{L}_{\mathrm{g}} ^{2}}{1-\mathcal{L}_{\mathrm{g}} } \biggr) \frac{\mathfrak{\mathrm{b}}\Gamma ({\mathfrak{\mathrm{\alpha }}}+1)+ \mathfrak{\mathrm{b}}^{\mathfrak{\mathrm{\alpha }}}}{\mathbb{M}({\mathfrak{\mathrm{\alpha }}}-1)\Gamma ({\mathfrak{\mathrm{\alpha }}}+1)} < 1. \end{aligned}$$

Also the conditions of Theorem 3 are satisfied. Hence, we also have $\mathcal{C}_{\mathrm{g}, \mathfrak{\mathrm{\alpha }}}\neq 1$. Thus the solution is both Hyers–Ulam and generalized Hyers–Ulam stable. In a similar way one can easily verify the conditions of Hyers–Ulam–Rassias and generalized Hyers–Ulam–Rassias stability.

## Conclusion

We have effectively achieved several necessary conditions describing the stability and existence hypotheses for a class of BVP including ($\mathcal{ABC}$) fractional derivative and integration. Under the consideration of fixed point theorems like Banach and Krasnoselskii, the necessary outcomes have been set up. Moreover, by an appropriate use of concepts from nonlinear analysis, some sufficient outcomes for various types of Hyers–Ulam stability have been developed. By giving appropriate examples, all the theoretical results have been testified. In future such type of analysis can be established for more general type BVPs involving the aforementioned derivative. The manuscripts [35–39] are useful for any further investigations.

## Data Availability

Data used in this research has been included within the paper.
